# Leveraging new methods for comprehensive characterization of mitochondrial DNA in esophageal squamous cell carcinoma

**DOI:** 10.1186/s13073-024-01319-2

**Published:** 2024-04-02

**Authors:** Xuehan Zhuang, Rui Ye, Yong Zhou, Matthew Yibo Cheng, Heyang Cui, Longlong Wang, Shuangping Zhang, Shubin Wang, Yongping Cui, Weimin Zhang

**Affiliations:** 1 Cancer Institute, Department of Oncology, Peking University Shenzhen Hospital, Shenzhen Peking University-the Hong Kong University of Science and Technology (PKU-HKUST) Medical Center; Institute of Cancer Research, Shenzhen Bay Laboratory, Shenzhen, Guangdong 518000 China; 2 Department of Psychiatry, Li Ka Shing Faculty of Medicine, The University of Hong Kong, Hong Kong, China; 3 The Department of Thoracic Surgery, Shanxi Cancer Hospital; Key Laboratory of Cellular Physiology of the Ministry of Education, Department of Pathology, Shanxi Medical University, Taiyuan, Shanxi 030001 China; 4 State Key Laboratory of Molecular Oncology, Beijing Key Laboratory of Carcinogenesis and Translational Research, Laboratory of Molecular Oncology, Peking University Cancer Hospital & Institute; Research Unit of Molecular Cancer Research, Chinese Academy of Medical Sciences, Beijing, 100142 China

**Keywords:** Mitochondrial DNA, Esophageal squamous cell carcinoma, Non-ref NUMTs, mtDNA copy number, mtDNA variants

## Abstract

**Background:**

Mitochondria play essential roles in tumorigenesis; however, little is known about the contribution of mitochondrial DNA (mtDNA) to esophageal squamous cell carcinoma (ESCC). Whole-genome sequencing (WGS) is by far the most efficient technology to fully characterize the molecular features of mtDNA; however, due to the high redundancy and heterogeneity of mtDNA in regular WGS data, methods for mtDNA analysis are far from satisfactory.

**Methods:**

Here, we developed a likelihood-based method dMTLV to identify low-heteroplasmic mtDNA variants. In addition, we described fNUMT, which can simultaneously detect non-reference nuclear sequences of mitochondrial origin (non-ref NUMTs) and their derived artifacts. Using these new methods, we explored the contribution of mtDNA to ESCC utilizing the multi-omics data of 663 paired tumor-normal samples.

**Results:**

dMTLV outperformed the existing methods in sensitivity without sacrificing specificity. The verification using Nanopore long-read sequencing data showed that fNUMT has superior specificity and more accurate breakpoint identification than the current methods.

Leveraging the new method, we identified a significant association between the ESCC overall survival and the ratio of mtDNA copy number of paired tumor-normal samples, which could be potentially explained by the differential expression of genes enriched in pathways related to metabolism, DNA damage repair, and cell cycle checkpoint. Additionally, we observed that the expression of *CBWD1* was downregulated by the non-ref NUMTs inserted into its intron region, which might provide precursor conditions for the tumor cells to adapt to a hypoxic environment. Moreover, we identified a strong positive relationship between the number of mtDNA truncating mutations and the contribution of signatures linked to tumorigenesis and treatment response.

**Conclusions:**

Our new frameworks promote the characterization of mtDNA features, which enables the elucidation of the landscapes and roles of mtDNA in ESCC essential for extending the current understanding of ESCC etiology. dMTLV and fNUMT are freely available from https://github.com/sunnyzxh/dMTLV and https://github.com/sunnyzxh/fNUMT, respectively.

**Supplementary Information:**

The online version contains supplementary material available at 10.1186/s13073-024-01319-2.

## Background

Mitochondria are essential organelles in human cells. The key function of mitochondria is to provide adenosine triphosphate (ATP) to cells through oxidative phosphorylation for various life activities [1]. It has therefore been implicated in carcinogenesis because the disruption of bioenergetic homeostasis is a common feature of cancers [2]. In addition, mitochondria extensively participate in biosynthesis, metabolism, and signal transduction and play important roles in controlling cell cycle, differentiation, and apoptosis, all of which are intrinsically linked to tumorigenesis and progression [3–5].

The function of mitochondria is largely programmed by mitochondrial DNA (mtDNA), which is a 16,569-bp, double-strand, and circular molecule that includes the noncoding D-loop region and the coding regions of 13 protein-coding genes, 22 tRNAs, and 2 rRNAs [6, 7]. Previous studies have shown that the mtDNA signatures, including variants, copy number (mtCN), and nuclear sequences of mitochondrial origin (NUMTs), are associated with many cancer types [8]. Specifically, supportive evidence such as the somatic presence of functional mtDNA variants in tumor tissues have been described in many cancers [9–13]. The reduction of mtCN in tumors relative to the adjacent normal was observed in most cancers, while the opposite trend was found in lung and lymph cancers [14, 15]. Further observations have uncovered the association between patient survival and mtCN in several cancer types [15]. Additionally, NUMTs were demonstrated to be activated in tumors [16, 17], and those located on the tumor-associated genes might lead to tumorigenesis by disrupting the gene function [18]. Together, these studies confirmed the crucial role of mtDNA in cancers. However, as the esophageal carcinoma (EC) samples included in the international consortium were almost adenocarcinoma, the knowledge about the molecular profile of mtDNA as well as their contribution to esophageal squamous cell carcinoma (ESCC) remains limited.

EC is a common gastrointestinal malignancy, ranking the seventh and sixth in global cancer incidence and mortality, respectively [19]. ESCC is the dominant histological subtype of EC and has the highest incidence in China [20]. Despite the great progress in clinical treatment and targeted therapies, the prognosis of ESCC is still unsatisfactory, largely due to the partially known molecular features underlying ESCC etiology [21, 22]. While the molecular characterization of nuclear DNA (nDNA) has been extensively explored [23, 24], little was known about the contribution of mtDNA to ESCC. Even though a few studies have implied the potential relevance of mtDNA in ESCC [25–30], they were largely limited by the sample sizes and the applied technologies such as target sequencing and real-time PCR, whereby the NUMTs and their derived false positive mtDNA variants (NUMT-FPs) were undetectable, impeding the comprehensive portrait of mtDNA in ESCC.

Whole-genome sequencing (WGS) is by far the most effective technology to fully characterize the molecular profile of mtDNA. However, tens of thousands of coverages of mtDNA in the regular WGS data result in extremely high redundancy and heterogeneity. In addition, the existence of sequencing errors (10^–4^–10^–2^) [31, 32] and polymerase chain reaction (PCR) errors (10^–7^–10^–5^) [33] further increases the difficulty of detecting mtDNA mutations; the available tools are very limited and have certain restrictions. For instance, MToolBox [34] does not apply to data with a coverage depth greater than 5000 × ; Mutect2 [35] and VarScan2 [36] are not suitable for detecting mutations with low variant allele fraction (VAF). In fact, due to the lack of protective proteins and damage repair mechanisms, mtDNA is the first sensor for the subtle changes in the cellular environment [5]. It is therefore essential to detect low-heteroplasmic mtDNA variants to reflect early changes in the organism’s environment before the onset or in the early stages of human diseases.

Another challenge is to identify the non-reference NUMTs (non-ref NUMTs) as well as the derived false positives (non-ref NUMT-FPs) that could confound the detection of mtDNA variants with low VAFs. The integration of mtDNA to nDNA is an inevitable result of endosymbiosis, and most of them were the products of long-term cellular evolution and have been recorded in the human reference genome (ref-NUMTs) [37]. By contrast, the recently occurred non-ref NUMTs, especially those present as somatic, have been demonstrated to affect the stability of nDNA and the expression of the corresponding genes in human diseases [16, 18]. Previous studies have proposed methods such as NUMTs-detection and dinumt for detecting non-ref NUMTs from WGS data [38, 39]; however, tools that can simultaneously detect non-ref NUMTs and non-ref NUMT-FPs were still lacking.

Here, we propose dMTLV for **d**etecting **mt**DNA variants with **l**ow **V**AF, and describe fNUMT, for **f**inding non-ref **NUMT**s, as well as their derived NUMT-FPs. In addition, we present a sample-specific filter strategy for mtDNA variants based on non-ref NUMT-FPs and mtCN. We then applied these methods to the WGS data of 663 paired ESCC tumor-normal samples. Coupled with the RNA-seq and whole-genome bisulfite sequencing (WGBS) of the 155 ESCC pairs therein, we present the most comprehensive multi-omics features of mtDNA in ESCC, extending the current understanding of ESCC etiology.

## Methods

### The likelihood-based method dMTLV

dMTLV performs two rounds of likelihood tests to reduce the noise of sequencing and PCR errors and generate highly confident mtDNA variants. dMTLV is available from https://github.com/sunnyzxh/dMTLV with the details described as follows.

dMTLV takes bam files as input and constructs read families based on chromosome coordinates and CIGAR strings of pair-end read. In a read family, for each position containing potential alternative alleles with the following criteria: (1) supported by both strands, (2) base quality > 20, and (3) mapping quality > 30, let ***X*** indicate all the alleles and ***θ*** denote the allelic fraction. For reads covering this coordinate, *x*_*i*_ denotes the allele on read *i* that belongs to {1,2…*N*}, with the corresponding sequencing error represented as *e*_*i*._ The probability mass function of ***X*** is *P*(*x*|***θ***), with the index parameter of ***θ*** = (*θ*_*A*_, *θ*_*C*_, *θ*_*G*_, *θ*_*T*_)^*T*^. The parameter space of ***θ*** is ***Ω***. Assuming g ∈ {*A*, *C*, *G*, *T*}, and *θ*_*g*_ represents the allelic fraction of *g*, which has *θ*_*g*_ ∈ [0, 1] and ∑*θ*_*g*_ = 1, we can get:

1 $$\begin{array}{lc}\mathit P\left({\mathit x}_{\mathit i}\vert\boldsymbol{\theta}\right)&=\mathit P\left(\mathrm{not\ error}\vert\mathit g\right)\cdot\mathit P(\mathit g)+\mathit P\left(\mathrm{error}\vert\mathrm{not}\mathit{\ g}\right)\cdot\mathit P(\mathrm{not}\mathit{\ g})\\&=\left(1-{\mathit e}_{\mathit i}\right){\mathrm\theta}_{\mathit g}+\frac{{\mathit e}_{\mathit i}}3\left(1-{\theta}_{\mathit g}\right)\end{array}$$

the log-likelihood function of (1) can be present as:

2 $$\mathrm\ell\left(\boldsymbol{\theta}\right)=\sum_{\mathit i=1}^{\mathit N}\text{log}\mathit P\left({\mathit x}_{\mathit i}\vert\boldsymbol{\theta}\right)=\sum_{\mathit i=1}^{\mathit N}\text{log}\left(\left(1-{\mathit e}_{\mathit i}\right){\mathrm\theta}_{\mathit g}+\frac{{\mathit e}_{\mathit i}}3\left(1-{\mathrm\theta}_{\mathit g}\right)\right),\mathit g={\mathit x}_{\mathit i}$$

with the likelihood ratio test written as:

3 $${\mathit t}_{\mathit g}=-2\left\{{\mathrm\ell}_0\left(\boldsymbol{\theta}\right)-{\mathrm\ell}_1\left(\boldsymbol{\theta}\right)\right\}\sim\mathrm\chi_1^2$$

For the scenario of *θ*_*g*_ = 0, which was on the margin of the parameter space, we applied a previously presented method to fit *χ*^2^ by adjusting the test of the general likelihood ratio [40]. In addition, we applied the Monte Carlo method to compare the *p* values estimated theoretically and empirically with the aim to explore the power of our model when the sequencing errors were uniformly distributed.

The number of free variables was two and three under the null and alternative hypothesis, respectively, the degree of freedom of the *χ*^2^ is 1, and the probability of *g* is:

4 $${\mathit P}_{\mathit g}=1-\text{cdf}\left({\mathit t}_{\mathit g}\right)$$

in which cdf(*x*) denotes the cumulative density function of the *χ*^2^. For *P*_*g*_ below the threshold *α*, the null hypothesis is rejected, and *g* is regarded as the alternative allele in the position.

Even though *P*_*g*_ is not the probability of *H*_0,*g*_ being true and *g* being a sequencing error, it is a good estimation for the error rate of *g* under *P*_*g*_ ≤ 10^–3^. Following this, those alleles with *P*_*g*_ ≤ *α* in both strands were reserved and the other alleles were substituted with “N”. Then the reads covering the position are assembled as several consensus sequences.

Next, for the alleles at a consensus sequence, let *P*_*c*_(*g*) represents the error rate of *g*. Assuming the reads covering the position were generated from *n* DNA templates, a coalescent method was applied to infer the PCR errors [41]. Theoretically, the frequency of a PCR error exponentially decreases as the PCR cycles represented as m increase. Thereby, the combined PCR error rate could be denoted as the probability of successfully detecting a PCR error with frequency ≥ 2^−*m*^/*n*, which is equal to or below:

5 $$1-{(1-\mathit{error}\;\mathit{rate}\;\mathit{per}\;\mathit{cycle})}^{2^{\mathrm m}-1}$$

where *e*_*pcr*_(*g*) indicates the coalescent PCR error rate and *P*_*pcr*_(*g*) is the overall PCR error rate of all alleles within the consensus sequence, we can have:

6 $${\mathit P}_\mathit{pcr}\left(\mathit g\right)\approx\mathbf n\ast{{\mathit e}_\mathit{pcr}\left(\mathit g\right)}^2$$

As the range of PCR fidelity is (10^–7^–10^–5)^, the product of *P*_*c*_(*g*) and *P*_*pcr*_(*g*) is approximately zero, and the combined base quality of *g* on the consensus sequence is as follows:

7 $$\mathit Q\left(\mathit g\right)=-10{\text{log}}_{10}\left({{\mathit P}_\mathit{c}\left(\mathit g\right)+\mathit P}_\mathit{pcr}\left(\mathit g\right)\right)$$

The *Q*(*g*) was then transferred to an ASCII character. The consensus sequences and the quality of each base were then sorted into a BAM file, of which each position containing potential alternative alleles went through another round of likelihood tests using Eqs. (1) to (3), and the final confident mtDNA variations, as well as the supportive information, were generated. For indels, the number of consensus sequences that support the existence of the indel (I + , D +) were counted at each position and the one with the highest supporting number was chosen and outputted.

### Generation of simulation data

Twenty single nucleotide variations (SNVs), five short insertions, and five short deletions were randomly generated and added to the Revised Cambridge Reference Sequence (rCRS, NC_012920) using a custom script. The two artifact-prone regions chrM:300–317 and chrM:16,180–16,193, as well as chrM:3107 which is “N”, were blacklisted when generating the simulated random variants [42]. Seven VAFs of 0.001, 0.005, 0.01, 0.05, 0.1, 0.25, and 0.5 were simulated under the average mtDNA coverage of 5000 × , 10,000 × , and 20,000 × , generating a total of 21 datasets. Specifically, simNGS (https://www.ebi.ac.uk/goldman-srv/simNGS/) was applied to generate the pair-end 150 bp sequencing reads. For instance, when generating the simulation data with VAF: 0.001 and Coverage: 5000 × , of which the mutated coverage is 5 × , the parameters and command used to produce the fasta files are as follows:

- cat mutated_rCRS.fa | simLibrary -x 5 -r 150 --seed 12 > chrM_mut_5x.fasta
- cat wide_rCRS.fa | simLibrary-x 4995 -r 150 --seed 12 > chrM_wide_4995x.fasta
- cat chrM_mut_5x.fasta chrM_wide_4995x.fasta > chrM_sim_5000x_mut5x.fasta
- simNGS -p "paired" -O "chrM_sim_5000x_mut5x_PE150" -o "fastq" -s 12 runfile chrM_sim_5000x_mut5x.fasta

Here, the runfile (s_1_0033.runfile), which was estimated by AYB to describe the distribution of cluster intensities and noise in real life by an Illumina machine, was downloaded from https://www.ebi.ac.uk/goldman-srv/simNGS/runfiles5/151cycPE/s_1_0033.runfile. Noted that the fragment IDs, e.g., “Frag_1”, was replaced with “mut_Frag_1” or “wide_Frag_1” for chrM_mut_5x.fasta or chrM_wide_4995x.fasta, respectively, to avoid reads ID conflict in the alignment process.

### Performance evaluation of dMTLV and comparison with other tools

Using the above simulation data, the performance of dMTLV was evaluated and compared with other three published tools MToolBox [34], Mutect2 (“mitochondrial mode” of the Genome Analysis Toolkit (GATK) v4.1.1.0) [35, 43], and VarScan2 [36] with default parameters unless otherwise stated. Specifically, when using MToolBox, we added “-d 500000” in line 248 of the script “assembleMTgenome.py”, which generated the pileup file, to allow a maximum number of 500,000 reads to be included for each position because the default -d of 8000 could miss a substantial fraction of sequencing reads. As MToolBox has implemented a workflow that used gsnap to map sequencing reads to chrM and realigned the chrM-mapped reads to the reference genome containing both nDNA and mtDNA to remove the ref-NUMTs derived reads, we used the MToolBox generated “OUT2-sorted.bam” as the input of dMTLV, Mutect2, and VarScan2.

True positive rate (TPR, also called sensitivity or recall), positive predictive rate (PPV, also called precision), and their harmonic mean F1 score were used to quantify the performance of the above four methods and determine the best strategy for integrating these methods to get the most comprehensive and accurate results. Specifically, TPR was calculated by the number of detected true positive variants against the total number of simulated variants. PPV was measured by the number of detected true positive variants against the total number of detected variants. F1 score is calculated as 2 × (PPV × TPR) / (PPV + TPR). In addition, we evaluated the Pearson correlation between the observed and the simulated VAF of each variant for each tool across different coverage.

### Framework and performance evaluation of fNUMT

fNUMT is available at https://github.com/sunnyzxh/fNUMT and the details were described as follows. Reads mapped to rCRS were aligned to reference genome containing nDNA and rCRS using bwa mem with parameter “-t 9 -K 12 -Y” [44], and only proper paired reads were retained. Junction reads with one portion mapped to autosome and the remaining part aligned to rCRS were extracted and clustered to locate the mtDNA segment coordinates as well as the autosomal insertion site. Specifically, the mapped position of read clusters with the first part soft-clipped and the second part mapped to rCRS with CIGAR string format of “xxSxxM” is nearly the start coordinates of the transferred mtDNA segment; in contrast, the “xxMxxS” read clusters could localize the end positions; and the breakpoints of the insertion site could be localized by the mapping coordinates of all junction reads. The discordant paired reads with one read mapped to rCRS and the other aligned to autosome were also counted as supportive information of the existence of the non-ref NUMTs. The homozygosity or heterozygosity of the non-ref NUMTs was estimated by the fraction of supportive reads against the mean coverage of the autosome.

Next, both the junction reads and the rCRS mapped reads of the discordant read pairs were assembled using CAP3 [45] to generate NUMT consensus contigs which were then mapped to rCRS using blastn [46] for verifying the mtDNA segment and identifying the mismatch bases.

To evaluate the performance of fNUMT, two tumor-normal paired samples from two ESCC patients with both short-read (150 bp, pair-end) and long-read (Nanopore) sequencing data were used. The library preparation, sequencing, and bioinformatics pipeline were described in our previous study [47]. The performance of fNUMT was compared with NUMTs-detection [38] and dinumt [39]. For dinumt, only the non-ref NUMTs classified as “PASS” and had mtDNA segment positions were retained for comparison.

We validated the non-ref NUMTs detected from short-read sequencing using the long-read data of the same sample as follows: (i) extracted the reads mapped around the estimated insertion sites (± 100 bp), (ii) manually inspected the reads in Integrated Genomics Viewer (IGV) [48] and the called non-ref NUMT was determined as false if no insertion marks existed (colored in purple with a number inside indicating the length of inserted sequences), and (iii) for those with insertion marks of comparable length against the estimated, extracted the insertion sequences and aligned them to the reference genome using blastn. We determined the called non-ref NUMTs to be true if the insertion sequences could map to the estimated positions of mtDNA.

### Multi-omics data of 663 ESCC patients and mtCN calculation

In this study, a total of 663 patients from Shanxi and Xinjiang provinces diagnosed with ESCC were included. None of them received any prior treatment when the tumor and matched normal samples were collected. All the samples were subject to WGS, wherein 155 were under additional WGBS and RNA sequencing. The detailed process for sample collection, DNA/RNA extraction, library preparation, sequencing, and basic data processing were described in our previous studies [23, 24]. To measure the effect of oxidative damage induced during acoustic shearing, we calculated the global imbalance value (GIV) score of G-T/C-A (an indicator of oxidative damage) for all samples using “Damage estimator” after sequencing alignment [49]. The mean GIV score of 1.04 indicated that our data showed almost no oxidative damage. The average autosomal sequencing coverage of WGS for normal and tumor samples was 29.6 × and 62.5 × , respectively. In this study, for each sample, all the clean sequencing reads were mapped to rCRS first and the rCRS-mapped reads were realigned to reference genome containing nDNA and rCRS using gsnap and bwa for removing/detecting ref-NUMTs and non-ref NUMTs and subject for mtDNA variants detection. The mean coverage of mtDNA for normal and tumor samples was 11,957.1 × and 11,108.2 × , respectively. The mtCN was then calculated as:

$$\mathrm{mtDNA \,coverage}/\mathrm{mean \,autosome \,coverage }\times (\mathrm{purity }\times \mathrm{ ploidy}+ (1 -\mathrm{ purity}) \times 2)$$

The purity and ploidy of tumor samples were inferred using ABSOLUTE [50] whereas for normal samples the purity and ploidy were set as 1 and 2, respectively.

The RNA sequencing data were mapped to the reference genome GRCh37/hg19 using STAR (v2.4.2a) [51] and the expression level was estimated by RSEM (1.2.29) [52] using uniquely mapped reads. The methylation level of a certain region was calculated as the mean of the fraction of reads supporting CG of each position using a custom script.

### mtDNA variants filter based on non-ref NUMT-FPs and mtCN

The VAFs of non-ref NUMT-FPs were estimated as 1/(1 + mtCN) and 2/(2 + mtCN) for heterozygous and homozygous non-ref NUMTs, respectively. For the initial mtDNA variants within the inserted mtDNA segment, those (1) were the identified mismatches, (2) with VAF near or below the inferred threshold (the difference of observed VAF to the threshold, divided by the threshold < 1), were supposed to be non-ref NUMT-FPs and were thereby removed. Of note, for non-ref NUMTs larger than 1 kb, the entire sequences of the inserted mtDNA segment may not be assembled successfully; therefore, the mismatches within the unassembled regions were undetectable. In this case, we only applied the above criteria (2) to remove the potential non-ref NUMT-FPs. The initial mtDNA variants that were inferred mismatches but not satisfied with the criteria (2) may result from both non-ref NUMT-FPs and the true mtDNA variants and thereby be retained. For all initial mtDNA variants, those with VAF below 1/mtCN were removed.

### mtDNA mutational spectrum and signature

The mutational spectrum of mtDNA variants was calculated using a custom script for both six different substitutions and 96 different profiles considering the nucleotide context. The R package “MutationalPatterns” and “NMF” (non-negative matrix factorization) was applied to extract the mutational signatures of somatic mtDNA variants and calculate the cosine similarity against Catalogue of Somatic Mutations in Cancer (COSMIC) single base substitution (SBS) signatures (v3.3-June 2022) [53].

### Public databases for annotation and comparison

mtDNA variants were annotated using ANNOVAR with the parameter of “-buildver GRCh37_MT -dbtype ensGene” [54]. All the mtDNA variants were formatted under HGVS nomenclature [55] and interpreted following the ACMG/AMP guidelines specific for mitochondrial variations [10]. We compared the SNVs and Indels results of our study with the MITOMAP database (https://www.mitomap.org/foswiki/bin/view/MITOMAP) [56]. Specifically, we download the mitochondrial general variants (VariantsControl; VariantsCoding) for comparing variant frequency including SNVs and Indels. The frequency of MITOMAP variants was derived from 56,910 GeneBank sequences and 78,504 Control Region sequences which are therefore good representations of the variants’ frequencies of the overall population. We download the somatic mutations (MutationsSomatic) and the curated disease-associated mutations (MutationsRNA; MutationsCodingControl; ConfirmedMutations) for comparison in order to identify the possible functional variants. Noted that we ignored the double/multiple bases substitutions (e.g., AT-CC; AGAA-AGAAAGAA) and inversions recorded in the above datasets.

For the comparison of non-ref NUMTs, we downloaded the 1637 non-ref NUMTs identified from 66,083 human genomes (supplementary table S1 [38]). Among these, we applied the 1601 non-ref NUMTs with complete information on mtDNA breakpoints (1610) and successful transfer from hg38 to hg19 (9 failed) for comparison. The population frequency of East Asians was used (column name: frequency (EastAsian)). In addition, we downloaded the 141 distinct non-ref NUMTs identified in 946 low-coverage WGS data from the 1000 Genomes Project (1KGP) phase 1 and an additional 53 high-coverage WGS data from the Human Genome Diversity Project (HGDP, Supplementary Data: ALL.dinumt.phase1.hgdp.final.numts.sites.vcf [39]). The population frequency of 1 KGP and HGDP was calculated by dividing the number of “SAMPLES” by 999 (946 + 53). The non-ref NUMTs from different studies with nuclear insertion positions within 200 bp were determined as shared.

### Statistical analysis

The correlation test was performed using Pearson or Spearman’s rank correlation test in R. The comparison between/among groups was performed using the Wilcox, *t*-test, or one-way ANOVA test. The survival analysis was conducted by R package survival and survminer using the Cox model. The *p* values were adjusted by the Bonferroni method in multiple tests.

## Results

### A new likelihood-based tool for heteroplasmic mtDNA variants detection

**Fig. 1:**
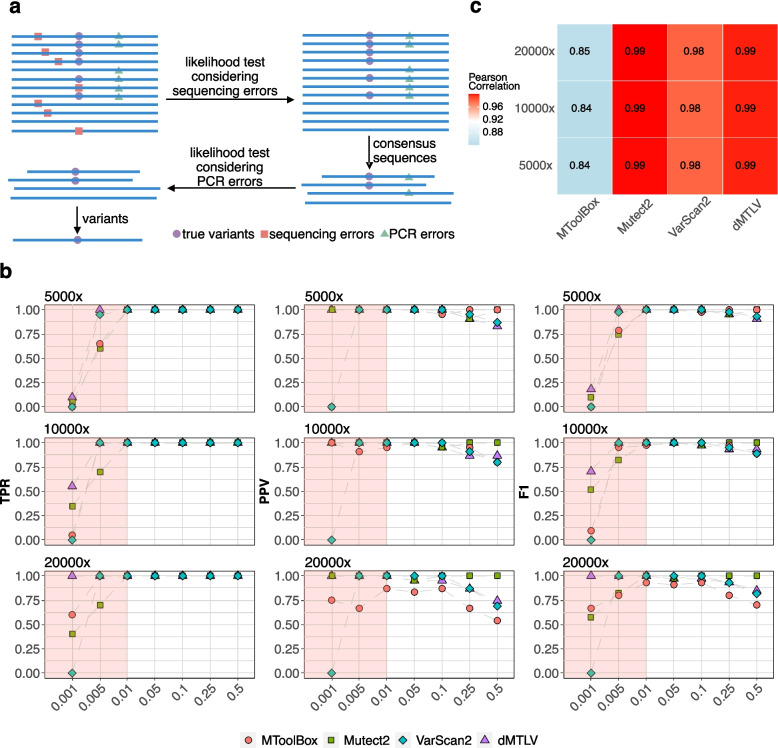
The workflow of dMTLV and the performance evaluation based on simulation data. **a** The workflow of dMTLV. For read family containing candidate alleles, dMTLV first performs a likelihood test to remove alleles likely to be sequencing errors, then constructs consensus sequences containing both true variants and PCR errors. Based on this, dMTLV performs a second likelihood test to eliminate PCR errors and generates the final result. **b** The TPR, PPV, and F1 of four tools: MToolBox, Mutect2, VarScan2, and dMTLV in detecting 20 randomly simulated SNVs under seven distinct VAFs and three different coverages. The comparison of VAF from 0.001 to 0.01 was highlighted with rectangles in light pink. **c** The Pearson correlation between the simulated and the observed VAFs by each tool across three different coverages. All the correlations were significant, with a *p*-value less than 0.01

We developed a new likelihood-based method named dMTLV for detecting mtDNA variants of low VAF from WGS data. Briefly, dMTLV first performs a likelihood test to remove sequencing errors, then constructs consensus sequences via local assembly, and conducts another round of likelihood tests to remove PCR errors, finally generating highly confident mtDNA variants (Fig. 1a, Method).

We assessed the performance of dMTLV using 30 randomly simulated mtDNA variants under seven VAFs of 0.001, 0.005, 0.01, 0.05, 0.1, 0.25, and 0.5 and three sequencing depths of 5000 × , 10,000 × , and 20,000 × . Three existing tools: MToolBox, Mutect2 (“mitochondrial mode”), and VarScan2 were used for comparison using the quantifying indicators of TPR, PPV, and their harmonic mean F1 score. The correlation between the observed and the simulated VAFs of each variant for each tool was also compared (Methods).

Specifically, dMTLV achieved the highest TPR, PPV, and F1 for VAF < 0.01 across all three coverages in simulation data, but the PPV started to decrease from VAF of 0.1, suggesting that dMTLV is appropriate for detecting ultra-low VAF below 0.01. MToolBox achieved an intermediate TRP, PPV, and F1 across all VAF when the average coverages were 5000 × and 10,000 × but had the lowest PPV and thus the lowest F1 when the coverage reached 20,000 × , which is consistent with the initial report in the paper proposing MToolBox that it is appropriate for data with relatively low coverage. VarScan2 had the lowest TPR, PPV, and F1 when the VAF was 0.001, but performed intermediately when the VAF was larger across all coverages. Similarly, Mutect2 performs poorly when the VAF is below 0.01, but achieves the best result when VAF > 0.01 for all coverages, which is in line with its original design to detect mutations with a VAF greater than 0.01 (Fig. 1b).

For short insertion and deletions, across all coverages, Mutect2 had generally the lowest TPR for VAF <  0.01 but achieved a consistently high PPV for VAF > 0.01 for all coverages. By contrast, VarScan2 had the lowest PPV for both insertions and deletions across all coverages and VAFs. VarScan2 performed poorly for indels, especially for insertions. dMTLV performed the second best after Mutect2 for both insertions and deletions for all coverages and VAFs (Additional file 1: Fig. S1).

Mutect2 achieved the highest Pearson correlation for the VAFs between the simulated and the observed variants, followed by dMTLV and VarScan2. MToolBox had the lowest correlation probably due to its discarding anomalous read pairs when generating pileup files (Fig. 1c).

Collectively, dMTLV improved the TRP without sacrificing PPV for detecting mtDNA variants with VAF < 0.01, and the improvement gets more significant as the sequencing coverage increases. This result suggested that the integration of mtDNA variants detected by Mutect2 with VAF > 0.01 and those detected by dMTLV with VAF from 0.001 to 0.01 could capture the most accurate mtDNA variants covering the most comprehensive VAF ranges.

### A new framework for non-ref NUMTs and NUMT-FPs detection

**Fig. 2:**
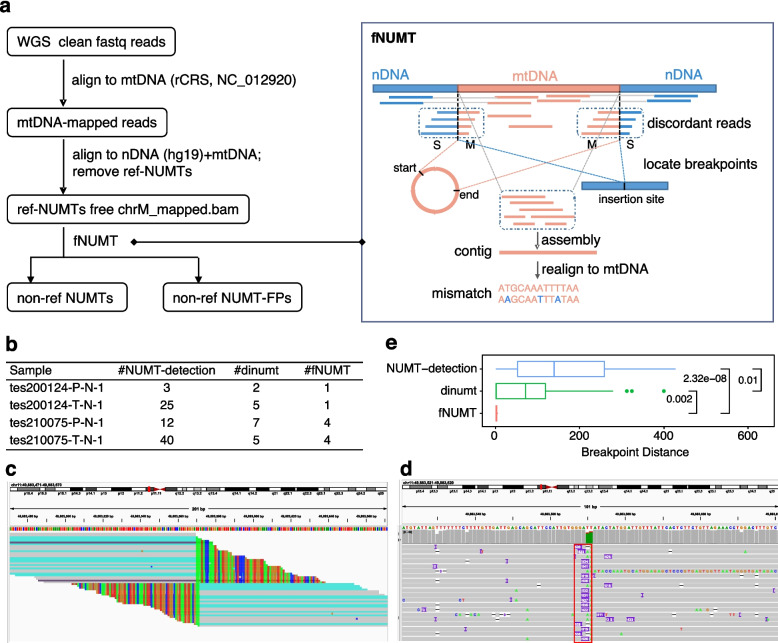
The workflow of fNUMT and the performance evaluation. **a** (Left) The preprocess of WGS data for downstream analysis. The WGS clean data were first aligned to rCRS. The mtDNA-mapped reads were then aligned to the reference containing nDNA and rCRS to remove the candidate ref-NUMTs covered by reads that were repetitively mapped to autosome and rCRS. The ref-NUMT free data were subsequently input to fNUMT for detecting non-ref NUTMs and NUMT-FPs. (Right) fNUMT searches the candidate junction reads with one end mapped to nDNA and the other to mtDNA. Then cluster such soft-clipped reads according to the mapping coordination and direction to locate the breakpoints of the inserted mtDNA segments and the insertion site on nDNA. Next, fNUMT assembly all the reads mapped to the candidate mtDNA segments and realign the contig to mtDNA to confirm the occurrence of the non-ref NUTMs and detect the mismatches that were the potential non-ref NUMT-FPs. **b** The number of non-ref NUMTs detected by NUMTs-detection, dinumt, and fNUMT in the four samples. **c, d** An illustration of the confirmation of the non-ref NUMTs by long-read sequencing data, taking the non-ref NUMT chrM:59–16089-chr11:49883569 as an example. **c** The IGV plot of short-read data near chr11:49883569 where the soft-clipped reads that could also map to mtDNA (colored in blue) were observed, meaning the presence of insertion sequences originated from mtDNA near chr11:49883569. **d** The IGV plot of long-read data near chr11:49883569 where the insertion of sequences with inferred sizes of ~ 530 bp (red box) were observed. **e** The distance of the breakpoints estimated by NUMTs detection, dinumt an fNUMT over those inferred by long-read data. The *p*-value was measured by the Wilcox test

We developed fNUMT for detecting non-ref NUMTs and the derived non-ref NUMT-FPs (Fig. 2a). Briefly, fNUMT takes the ref-NUMTs free bam file as input, of which the reads repetitively mapped to both nDNA and mtDNA were removed and extracts the soft-clipped reads with one portion of sequence mapped to nDNA and the remaining to mtDNA. These potential junction reads were then clustered to locate the insertion site of nDNA and the start and end positions of the inserted mtDNA segments. The latter is essential as the mtDNA are circular molecular with artificially defined coordination, unlike the segments in nDNA, of which the numerically smaller coordinate was naturally taken as the start position, the segments spanning the artificial break in the circular genome had a start position numerically larger than the end position. Nevertheless, the start and end positions were ambiguous in most previous studies, which confused the determination of segment size and the further exploration of the consequences of the non-ref NUTMs.

Next, fNUMT extracted and assembled the mtDNA-mapped sequences either from the potential junction reads or the discordant read pairs with one read mapped to mtDNA and the other to nDNA. The consensus sequence was then mapped to rCRS and the mismatched were detected as the potential non-ref NUMT-FPs.

To evaluate the performance of fNUMT, we applied it to the short read (150 bp, pair-end) WGS data of two ESCC paired tumor-normal samples and verified the results using the Nanopore long-read WGS data of the same samples [47]. The performance of fNUMT in detecting non-ref NUMTs was compared to the published methods NUMTs-detection [38] and dinumt [39]. The mean mtDNA coverage of these four samples was ~ 11,054, which is approximately five times higher than that of ~ 2000 × in the NUMTs detection paper. Therefore, when applied NUMTs-detection in our data, proportionally, we set the minimum supported discordant reads to 25 from the 5 used in the NUMTs-detection paper [38].

Specifically, the number of non-ref NUMTs detected by fNUMT, NUMTs-detection, and dinumt differed in all four samples (Fig. 2b). fNUMT identified one and four germline non-ref NUMTs for each patient, respectively. We verified all the ten non-ref NUMTs by the long-read sequencing data wherein we observed the insertion of sequences with inferred sizes near the estimated breakpoint of nDNA, and the inserted sequences could map to the coordinates of the inferred mtDNA segment (Fig. 2c, d, Additional file 1: Fig. S2, Additional file 2: Table S1). Of note, two non-ref NUMTs verified by long-read data were not detected by NUMTs-detection and dinumt, indicating that fNUMT had better specificity. Moreover, we ranked the non-ref NUMTs identified by NUMTs detection and dinumt according to the confidence decided by the number of supported signals and the quality score, separately, and observed that even the most confident one cannot be confirmed by the long-read sequencing data (Additional file 1: Fig. S3).

Taking the breakpoints of long-read data as the gold standard, we next evaluated the precision of breakpoint estimation of fNUMT, NUMTs-detection, and dinumt using the eight shared non-ref NUMTs. Each non-ref NUMT had three (or four if the estimated two nDNA positions were not the same) breakpoint distances calculated by the absolute differences between the breakpoint estimated by each tool and the long-read data. Overall, fNUMT predicted the most precise breakpoints. The mean breakpoint distances of fNUMT, dinumt, and NUMTs-detection were 2.31 bp, 88.63 bp, and 154.56 bp, respectively (Fig. 2e). Take the non-ref NUMTs: chrM:59–16089-chr11:49883569 in tes200124-T-N-1 as an example, of which the inferred mtDNA segments by fNUMT was chrM:61–16089, the same with that estimated by long-read data while the chrM:16090–16489 inferred by NUMTs-detection missed chrM:1–61 and chrM:16490–16569. In terms of the insertion site on nDNA, the chr11:49883569–49883572 estimated by fNUMT was 0 bp and 2 bp away from the long-read decided breakpoint of chr11:49883569 while the chr11:49883298–49883856 by NUMTs-detection were 271 bp and 287 bp away, and chr11:49883500–49883665 by dinumt were 69 bp and 97 bp away. More specifically, among the inferred 30 breakpoints of all 10 non-ref NUMTs detected by fNUMT, 11 were the same, 10 with the distance of 1 bp, 4 with 2 bp, 2 with 3 bp, and 2 with 6 bp, indicating that the breakpoints inferred by fNUMT were very close to that identified by long-read sequencing data (Additional file 2: Table S1).

In addition, fNUMT identified 22, 24, 28, and 27 potential non-ref NUMT-FPs in each of the four samples, among which two were indels present in all samples (m.16258_16259insA and m.16264_16264del) that accumulated in the non-ref NUMT chrM:61–16089-chr11:49883569–49883572. As non-ref NUMT-FPs were not generated by NUMTs-detection and dinumt, the performance regarding non-ref NUMT-FPs could not be compared.

### Multi-omics data of ESCC and the mtCN characterization

**Fig. 3:**
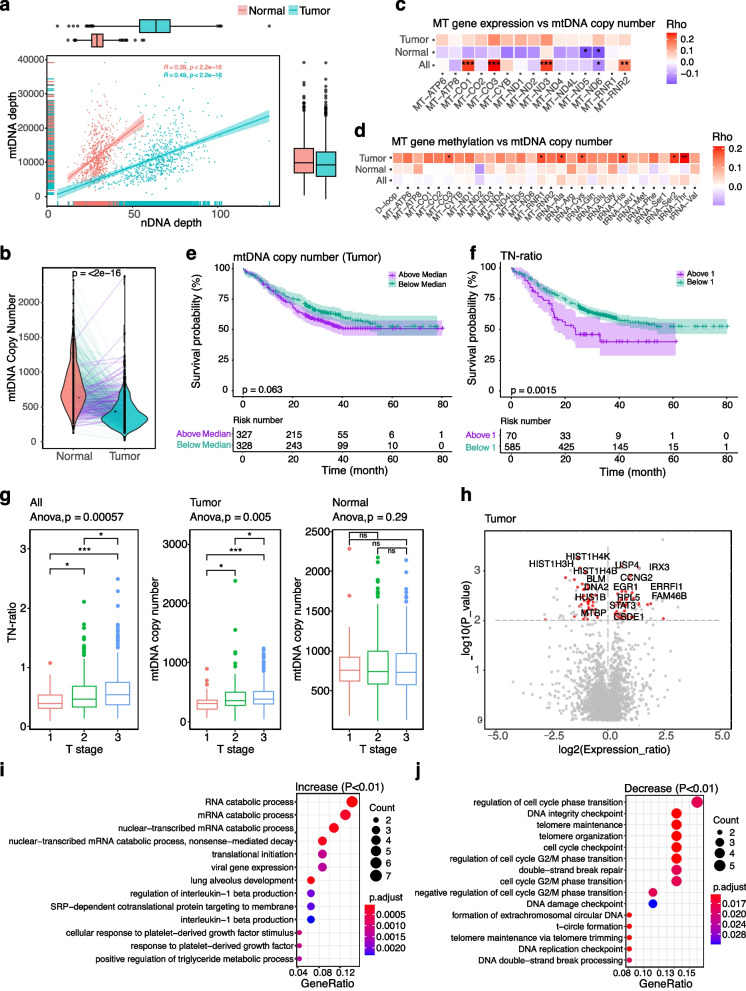
The multi-omics feature of mtCN in 663 ESCC paired tumor-normal samples. **a** The sequencing coverage of mtDNA and nDNA in the 1326 patients. The correlations were evaluated by the Pearson test. **b** The mtCN in normal and tumor samples. Lines colored in blue indicate a lower mtCN in the tumor over the paired normal samples, otherwise in purple. The *p*-value was calculated by the two-sided *t*-test. **c** The Spearman correlation between the expression of the mtDNA coding genes with mtCN in the tumor, normal, and all samples. **d** The Spearman correlation between the methylation level of the mtDNA regions with mtCN in the tumor, normal, and all samples. **e** Plots of overall survival for ESCC patients with mtCN above and below the median value. **f** Plots of overall survival for ESCC patients with TN-ratio above and below 1. For both **e** and **f**, the shaded areas represent the 95% confidence intervals, with the risk table under the survival plot. *p* values were calculated by log-rank test. **g** The TN-ratio and mtCN under different T stages. The pairwise comparison was performed by the Wilcox test (* < 0.05; ** < 0.01; *** < 0.001; **** < 0.0001, ns not significant), and the overall *p* values were calculated by the one-way ANOVA test. **h** The differentially expressed genes of the tumor samples of patients with TN-ratio > 1 over those with TN-ratio < 1. *p* values were calculated by the Wilcox test and adjusted by the Bonferroni method. **i, j** The enriched GO terms of genes significantly (*p* < 0.01) upregulated (**i**) and downregulated (**j**) in the tumor samples of patients with TN-ratio > 1. The *p* values were adjusted by the Bonferroni method

In this study, we included the WGS data of 663 ESCC paired tumor-normal samples, of which the RNA-seq and WGBS were also available in 155 patients [23, 24]. The mtCN was estimated by the sequencing depth of mtDNA (normal: 11,957.1 × , tumor: 11,108.2 ×) and nDNA (normal: 29.6 × , tumor: 62.5 ×), which showed a significant positive correlation in each sample type (Fig. 3a). We further adjusted the mtCN in tumor samples based on the purity and ploidy estimated by ABSOLUTE [50]. On average, the mtCN of tumor samples was 412.3, significantly lower than 808.5 in normal samples (*p* < 2e − 16). At the individual level, 592 out of 663 (89.3%) patients had decreased mtCN in the tumor compared to the paired samples, while the remaining 71 patients had the opposite trend (Fig. 3b).

We next evaluated the relevance of mtCN to gene expression and methylation. Specifically, we found a significant positive correlation between the expression of *MT-CO1, MT-CO3, MT-ND3,* and *MT-RNR2* with mtCN in all samples, while a negative association was found in *MT-ND6* (Fig. 3c). Regarding the methylation level of all mtDNA regions, we observed a significant positive correlation of the methylation level of *MT-CO3*, *MT-RNR1*, and five tRNAs (particularly tRNA-Thr) with mtCN in tumor samples; however, this was not found in normal and all samples (Fig. 3d).

Previous mtDNA studies in pan-cancer have shown that a high mtCN in tumor tissue is associated with a better prognosis in some cancer types and an inverse trend in others [14]. In this study, the patients with high mtCN in tumor samples tended to have worse overall survival; however, this was only marginally significant (*p* = 0.063, Fig. 3e).

Instead, we observed a significant association (*p* = 0.0015) between the overall survival with the TN-ratio calculated as the ratio of mtCN between the paired tumor and normal sample (Fig. 3f). Specifically, patients with TN-ratio > 1 had worse overall survival, suggesting that the tumor cells in these patients may have a better ability to compete for individual energy than normal cells, thus leading to a worse prognosis. Given the differences in energy metabolism basis between individuals, the comparison of TN-ratio would be more reasonable than simply comparing the mtCN of tumor tissues among patients.

Interestingly, we observed that the TN-ratio increased with the T stage progressed (*p* = 0.00057), which was mainly ascribed to the increase of mtCN in tumor samples (*p* = 0.005), while the mtCN was nearly unchanged in normal samples (Fig. 3g). This result was similar to the previous finding that mtCN increased as diseases progressed from noncancerous esophageal mucosa to ESCC and finally to lymph node metastasis [25]. Since patients with high T stage tend to have a worse prognosis, this result supported the correlation between TN-ratio and overall survival from another perspective. Except for the T stage, we did not observe significant associations between TNratio with other clinical features.

We further explored the genome-wide differentially expressed genes between patients with TN-ratio > 1 (group 1, patient number: 19) and those with TN-ratio < 1 (group 2, patient number: 136; Fig. 3h). We observed that the up-regulated genes in group 1 were enriched in energy metabolism-related pathways, suggesting that the tumor cells in these patients had better metabolic capacity (Fig. 3i). On the contrary, many of the down-regulated genes are histone modification-related genes. As some of the intermediates of mitochondria are the substrates of chromatin modification enzymes in the nucleus, the down-regulated expression of histone modification-related genes may be caused by the regulation of mitochondrial intermediates, which may increase chromatin accessibility and thus promote the proliferation of tumor cells. Other down-regulated genes were significantly enriched in cell cycle checkpoint and DNA damage repair-related pathways (Fig. 3j), suggesting that the tumor cells in group 1 were more capable of escaping the cell cycle monitoring and damage repair, which would be relevant with the malignant proliferation of tumor cells which thus lead to worse survival.

### The profile of non-ref NUMTs in ESCC

**Fig. 4:**
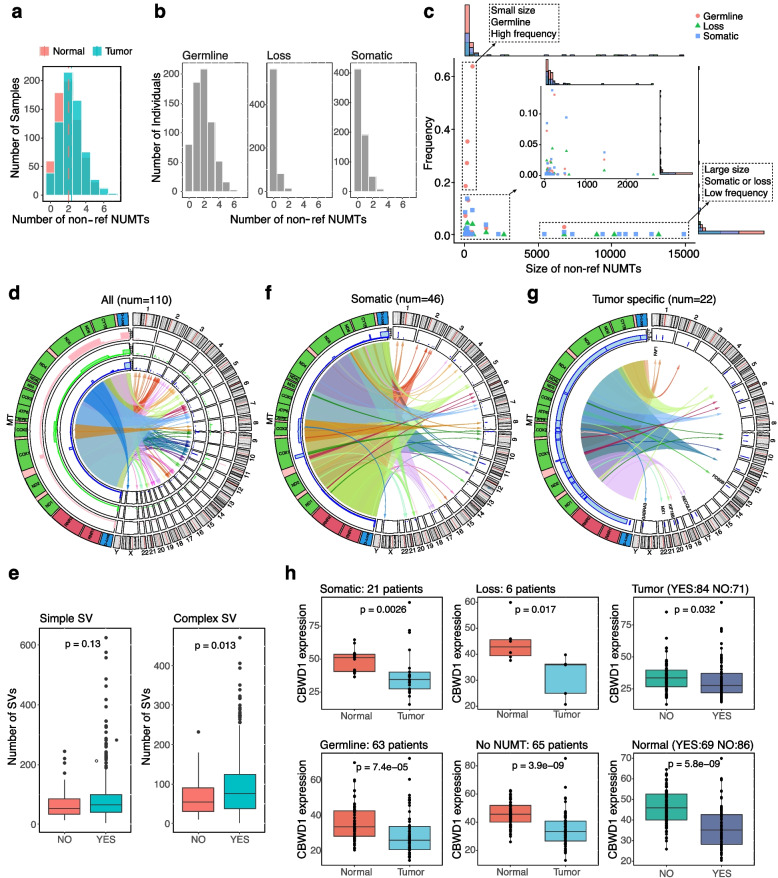
The landscape of non-ref NUMTs in 663 ESCC patients. **a** The number of non-ref NUMTs per sample. The dashed lines indicate the average number of non-ref NUMTs per sample type. **b** The number of non-ref NUMTs of the germline, loss, and somatic per individual. **c** The correlation between the frequency and size of the 110 distinct non-ref NUMTs detected in all 1326 samples, with summary histograms at the edges. The high-frequent non-ref NUMTs may include more than one type of source indicated by different colors and shapes. The distribution of non-ref NUMTs with frequency < 0.2 and size < 2500 bp was zoomed in. **d** The circos plot of 110 distinct non-ref NUMTs. From the outside: (1) mtDNA genes (left) and nDNA chromosome (right); (2) frequencies of non-ref NUMTs reported in Wei W. et al. [38]; (3) frequencies of non-ref NUMTs reported in Dayama G. et al. [39]; (4) frequencies of non-ref NUMTs detected in this study; and (5) arrows representing the insertion of mtDNA segments to the breakpoints of nDNA. **e** The number of somatic simple (left) and complex (right) SVs in 528 tumor samples with (num = 468) and without (num = 60) non-ref NUMTs. *p* values were calculated by the Wilcox test. **f** The circos plot of 46 distinct somatic non-ref NUMTs. **g** The circos plot of 22 tumor specific non-ref NUMTs. The genes with non-ref NUMTs on the intron region were labeled. Only the frequency in our cohort was shown in **f** and **g**. **h** (Left, middle) The expression of *CBWD1* in different sample types in patients with different sources of the non-ref NUMTs chrM:6225–6420-to-chr9-129767. (Right) The expression of *CBWD1* in samples with and without the non-ref NUMTs. *p* values were calculated by the Wilcox test

We applied fNUMT to detect the non-ref NUMTs of 663 ESCC paired tumor-normal samples based on the short-read WGS data. At the sample level, the average number of non-ref NUMTs in normal samples was 2.1, slightly lower than 2.4 in tumor samples (Fig. 4a). Overall, 1227 (92.53%) had at least one non-ref NUMTs. More specifically, 1166 (87.93%) had at least one germline non-ref NUMTs; 249 (40.62%) tumor samples had at least one somatic non-ref NUMTs, and 98 (15.99%) patients had at least one non-ref NUMTs present in normal sample but absent in the paired tumor sample (loss) (Fig. 4b).

At the population level, we identified a total of 110 distinct non-ref NUMTs, 85 (77.3%) of which were < 1 kb and present in < 10% of 663 ESCC patients (Fig. 4c). On average, the size of non-ref NUMTs in tumors (470.47 bp) was slightly larger than in normal samples (443.26 bp). Interestingly, we observed that the most frequent four non-ref NUMTs had small sizes (< 100 bp), and all were present as germline. On the contrary, the 14 non-ref NUMTs with the largest sizes had low frequencies (< 5%) and were mostly (13, 92.9%) somatic or loss. These results implied that the non-ref NUMTs may have potential roles in ESCC and thereby underwent different selection pressures.

We also compared the 110 non-ref NUMTs with those (num = 1601, Methods) identified in 66,083 human genomes from 100,000 Genomes Project in England [38], and in 946 and 53 genomes from 1 KGP and HGDP, respectively [39] (Fig. 4d). Generally, the insertion sites of the 110 distinct non-ref NUMTs were distributed across the whole genome; the most frequent non-ref NUMTs (> 10%) occurred on chr11, chr9, chr4, chr3, chr2, and chr1, with corresponding mtDNA region of D-loop, *MT-COX1*, *MT-ND5*, and *MT-CYTB* (shared by the last three), which was similar with previous finding. Most (94, 85.5%) of the non-ref NUMTs were < 1 kb (Additional file 1: Fig. S4). Overall, 24 non-ref NUMTs were also identified previously. When ranking the 110 non-ref NUMTs according to the population frequency, all the top 13 non-ref NUMTs (> 10%) were also found in previous studies with comparable population frequencies (Additional file 2: Table S2). The most frequent non-ref NUMT, present in 69.83% of samples, was the insertion of the 541 bp D-loop segment (chrM:16089–61) spanning the artificial break to chr11:49883569, which would otherwise be mistaken as chrMT:61–16089 with the size of 16,027 if the start and end positions were not accurately determined.

Noteworthy that one non-ref NUMT (chrM:12714–14830-to-chr5:32338477 spanning *MT-ND5*, *MT-ND6,* and *MT-CYTB*) identified previously with the East-Asian frequency of 68% and the 1 KGP and HGDP frequency of 25.93% was absent in our cohort (Fig. 4d). This non-ref NUMT was also detected by NUMTs-detection and dinumt in the two ESCC paired samples used for fNUMT performance evaluation. However, as indicated by the long-read sequencing data, the size of the sequence inserted to chr5:32338477 was 293 bp rather than 2116 bp (Additional file 1: Fig.S5). Furthermore, this 293 bp sequence was the “Homo sapiens chr5:32338477–32338478 non-reference unique insertion sequence” (NCBI accession number: MH534373), of which the 1–147 bp aligned to chrM:14832–14987, and the 147–190 bp reversely aligned to chrMT:12723–12867, leading to the wrong identification of the insertion of chrM:12714–14830. By contrast, fNUMT managed to avoid such false positives by clustering potential junction reads according to their mapping orientation and filtering out candidate non-ref NUMTs with orientation conflict or only one type of orientation cluster.

### Potential consequences of non-ref NUMTs in ESCC

We next investigated the association of non-ref NUMTs with the previously dissected somatic structural variants (SVs) in the nDNA of 528 ESCC individuals (out of the 663 in this study) [47]. Interestingly, we observed that the tumor samples carrying at least one non-ref NUMTs (num = 468) tend to have more SVs that were defined as complex than those without any non-ref NUMTs (num = 60, *p* = 0.013, Fig. 4e), implying that the insertion of mtDNA sequences to nDNA may affect the stability of nDNA thus leading to complex SVs. As expected, this trend was not found for simple SVs, meaning that the initiation of simple SVs was not affected by non-ref NUMTs (Fig. 4e).

Regarding the origins of non-ref NUMTs, we identified 46 distinct somatic non-ref NUMTs with a mean size of 952.45 bp (Fig. 4f). The most frequent somatic non-ref NUMTs (present in 85 patients) was the insertion of 195 bp segments in *COX1* to the intronic region of *CBWD1* (chrM:6225–6420-to-chr9-129767). Among the somatic non-ref NUMTs, 22 (present in 23 patients) were ESCC tumor-specific absent in any normal samples of our cohort and the previous study [38]. Five such non-ref NUMTs were in the intron region of *FAF1, PDS5B, RECQL5, KIF16B*, and *GABRA3*, and 1 in the UTR3 of *MX1* (Fig. 4g).

Among the 75 germline non-ref NUMTs (Additional file 1: Fig. S5), the one on the intronic region of *TCF12* (chrM:7179–7295-to-chr15:57220850, present in one individual) would be interesting as *TCF12* is a known tumor-associated gene (COSMIC databases, [53]). Additionally, as the insertion of mtDNA to the exon region is very rare, one germline non-ref NUMT (chrM:11707–11854-to-chr8-139661937, present in one individual) on the exon region of *COL22A1* would also be appealing.

We next explored the potential effect of non-ref NUMTs on the expression of the host or nearest genes. We evaluated the 11 non-ref NUMTs present in at least 5 samples of 155 patients with available RNA-seq data. The result showed that the non-ref NUMT chrM:6225–6420-to-chr9-129767 on the intron region of *CBWD1*, present in 153 out of 310 samples (21 somatic, 126 germlines, and 6 loss), downregulated the expression of *CBWD1* particularly in normal samples (Fig. 4h). Regardless of whether this non-ref NUMT exists, the expression of *CBWD1* was significantly lower in tumors (34.24) than in normal samples (42.09). However, this non-ref NUMT further reduced the expression of *CBWD1* in normal samples from 46.36 to 36.75, with the latter comparable to that of tumor samples. Moreover, the expression of *CBWD1* was further reduced in tumor samples with the non-ref NUMTs. In fact, CBWD1 enables ATP binding activity and is active in the cytoplasm [57], which may be the reason of the high frequency of this non-ref NUMT. The downregulation of *CBWD1* may affect the ATP binding activity and the subsequent ATP production, which may modulate the cellular energy metabolism, and thereby providing conditions for tumor cells to adapt to a hypoxic environment.

### The landscape of mtDNA variants in ESCC

We applied dMTLV and Mutect2 to detect mtDNA variants of the 663 ESCC paired tumor-normal samples from the ref-NUMT free bam files described in Fig. 2a. We obtained an average of 1070 initial mtDNA variants per sample by integrating mtDNA variants with VAF > 0.01 detected by Mutect2 and those with VAF from 0.001 to 0.01 detected by dMTLV. In addition, we identified an average of 76 potential non-ref NUMT-FPs (Additional file 1: Fig. S6; Method) for each sample. After removing non-ref NUMT-FPs, we observed a strong negative correlation between the number of mtDNA variants with VAFs ranging from 0.001 to 0.01 and mtDNA coverage (*R* =  − 0.34, *p* < 2.2e − 16, Additional file 1: Fig. S7), suggesting the existence of depth-related false positive variants among these low-frequent mtDNA variants. As the variants should theoretically appear in at least one mitochondrion, those with VAF below 1/mtCN were supposed to be false positives. After removing these variants, the number of variants with VAF from 0.001 to 0.01 was in proportion with mtDNA coverage (*R* = 0.26, *p* < 2.2e − 16, Additional file 1: Fig. S7), consistent with the rationale that the increase of sequencing coverage improves the chance of identifying low-frequent altered alleles. As expected, the detection of mtDNA variants with VAF above 0.01 was rarely affected by mtDNA coverage, indicated by the similar R score before and after mtCN-based filtering (Additional file 1: Fig. S7).

**Fig. 5:**
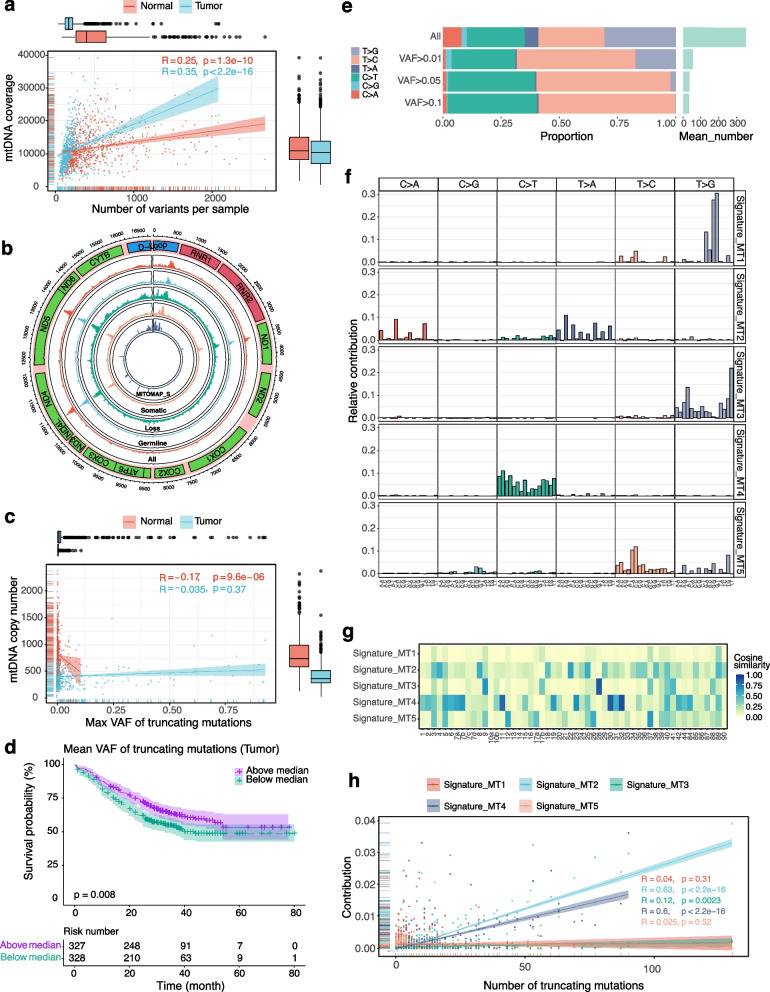
The profiling of mtDNA variants in ESCC. **a** The correlation between the number of mtDNA variants and coverage in normal and tumor samples, with the summary boxplot in the margin. The relation coefficients and *p* values were calculated by the Pearson test. **b** The circos plot of the population frequency of the 24,347 distinct mtDNA variants detected in the 1326 samples. From the outside: (1) the position of the mtDNA; (2) the regions of mtDNA; (3) the frequency of all, germline, loss, and somatic mtDNA variants; and (4) the frequency of somatic mtDNA variants in the MITOMAP database. **c** The correlation between the max VAF of truncating mutations with mtCN in tumor and normal samples, with the summary boxplot showing in the margin. The relation coefficients and *p* values were calculated by the Pearson test. **d** Plots of overall survival for patients with mean VAF of truncating mutations of tumor sample above and below the median value. The colored areas indicate the 95% confidence intervals, with the risk table under the survival plot. *p* value was measured by log-rank test. **e** The proportion of six mutational types under different VAFs, with the mean number per type shown on the right. **f** Five mutation signatures extracted from the somatic mtDNA variants. **g** The cosine similarity between the five signatures in our cohort with COSMIC single base substitution signatures. **h** The correlation between the number of truncating mutations with the contribution of the five mutation signatures. The relation coefficients and *p* values were calculated by the Pearson test

The above pipeline of mtDNA variants detection and filtering resulted in an average of 214.5 variants in tumors, significantly lower than 521.9 in normal samples (*p* < 2e − 16, Fig. 5a), consistent with the hypothesis that tumor cells were able to eliminate defective mitochondria (with mtDNA variants) to keep energy homeostasis to survive the changing micro-environment during tumor initiation and progression [58]. Additionally, the number of mtDNA variants was in proportion with mtDNA coverage, suggesting the robustness of the mtDNA variants.

At the population level, we identified 24,347 distinct mtDNA variants. The hotspot regions include D-loop, *MT-ND1*, *MT-ND4*, and *MT-ND5* regardless of the source of the variants (germline, loss, or somatic, Fig. 5b). Among these, 10,316 (42.4%) variants were also reported in MITOMAP database (the overall population cohort), and 700 (2.88%) were previously identified as disease-associated variants, of which 73 were experimentally confirmed. Interestingly, 640 (2.63%) were reported as somatic variants of many cancer types wherein three variants in D-loop (215A > G, 16304 T > C, 16324 T > C) have been observed in EC. The somatic variants identified in our cohort and those found in cancers of MITOMAP both showed enrichment on D-loop and *MT-ND4*, while our results were additionally enriched on *MT-ND1* and *MT-ND5* (Fig. 5b). Additionally, we identified 126 ESCC tumor-specific variants (62 SNVs, 49 short insertions, and 15 short deletions) present in at least two tumors but not in any normal samples nor MITOMAP general populations (Additional file 2: Table S3).

We next evaluated the potential contribution of truncating mutations (stop-gain and frameshift indels) to ESCC. We observed that the per-sample max VAF of truncating mutations (t-VAF) in tumors was significantly higher than in normal samples (mean: 0.044 versus 0.0068, *p* = 2.25e − 19). Moreover, samples with max t-VAF above 0.11 were all tumors (num = 55), implying that tumor cells were more capable of accumulating mtDNA variants that were functionally advantageous while eliminating the damaged mtDNA, with the latter indicated by the reduced mtCN in tumors (Fig. 5c). More interestingly, we observed that patients with higher mean t-VAF in the tumor samples had better overall survival (*p* = 0.008, Fig. 5d), suggesting that tumors with more comprehensive elimination of dysfunctional mtDNA tended to have better survival.

The primary mutation types of mtDNA SNVs in our cohort were T > G, T > C, and C > T, with the mean proportion of 32.14%, 27.83%, and 23.89%, respectively (Fig. 5e, Additional file 1: Fig. S8, Additional file 1: Fig. S9a), which was different with other cancer types reported in previous studies wherein the C > T and T > C had the first and second largest proportion and T > G account only for a small proportion [14]. As this previous study only reported mtDNA variants with VAF > 0.01, we further analyzed the mutation types under different VAFs and observed that the mutation types of variants with VAF > 0.01 were also dominant by C > T and T > C, and the percentage continued to increase for variants with VAF > 0.05 and 0.1 (Fig. 5e). Interestingly, we observed worse overall survival for patients with a higher number of low-frequent T > G variants in tumor samples (*p* = 0.01, Additional file 1: Fig. S9b), indicating that these large percent T > G variants in our cohort may reflect the instability of mtDNA and thereby affect the prognosis of ESCC.

We next investigated the mutational signature of all somatic variants, which could be classified into five distinct signatures by the NMF method (Fig. 5f). The cosine similarity against COSMIC single base substitution (SBS) signatures (v3.3-June 2022) showed that Signature_MT3 significantly correlated with SBS28 (unknown) with a cosine similarity of 0.857, Signature_MT4 associated with SBS32 (Azathioprine treatment), SBS30 (Defective DNA base excision repair due to NTHL1 mutations), and SBS11(Temozolomide treatment), with similarity of 0.887, 0.839, and 0.805, respectively; Signature_MT2 marginally correlated with SBS22 (aristolochic acid exposure) with similarity of 0.594 and Signature_MT5 marginally associated with SBS37 (unknown) with similarity of 0.635. Signature_MT1 was supposed to be novel without obvious correlation with any COSMIC SBS signatures (Fig. 5g). Interestingly, we found that the number of truncating mutations was in proportion with the contribution of Signature_MT2 and Signature_MT4 (Fig. 5h), reflecting the potential association of mtDNA truncating mutation with aristolochic acid exposure, DNA base excision repair, azathioprine, and temozolomide treatment, all of which were relevant to tumorigenesis and treatment response.

## Discussion

In this study, we describe two new methods dMTLV and fNUMT to overcome the current challenges in mtDNA study, which could be broadly applied to the accumulated large-scale short-read WGS data to investigate the contribution of mtDNA to human diseases. Leveraging the new methods, we provide the most comprehensive characterization of mtDNA in ESCC and uncover some potential ESCC-associated biomarkers. Importantly, this work could serve as a paradigm for the mtDNA study of other phenotypes.

The dMTLV is distinctive from the existing state-of-the-art mtDNA variants detection tools in its utilization of a likelihood-based model and construction of consensus sequences for reducing the noise of sequencing and PCR errors, which achieved the best performance for detecting mtDNA variants with VAF < 0.01. Such low-heteroplasmic mtDNA is essential for investigating the functional mutations present before the onset or in the early stage of diseases. Indeed, the mtDNA variants with VAF < 0.01 account for a large proportion (81.8%) of all mtDNA variants in our cohort. The growth in mtDNA variants allowed for signature mining of the mutational spectrum to explore the underlying mechanism of disease etiology, which was unanalyzable in previous studies [14]. Of note, the reads repetitively mapped to both mtDNA and nDNA reference genomes should be discarded to remove the artifacts introduced by ref-NUMTs before being subjected to dMTLV. As dMTLV depends heavily on the result of reads alignment, the improvement in reads mapping algorithms in the future would also facilitate the performance of dMTLV.

The detection of low-heteroplasmic variants was also confounded by mtCN and non-ref NUMT-FPs, i.e., given the same VAF, variants in samples with lower mtCN have fewer supportive reads and are therefore less credible (Fig. 1b), and the variants accumulated on non-ref NUMTs in the nDNA were often mistaken as mtDNA variants due to reads misalignment. As a consequence, the previous mtDNA study of pan-cancer or large-scale cohorts only reported the most confident mtDNA variants with VAF > 0.01 [14] or even 0.1 [42], limiting the comprehensive profiling of the landscape of mtDNA variants. Here, we propose that the pipeline integrating dMTLV and Mutect2, coupled with the sample-specific filtering strategy for excluding non-ref NUMT-FPs and coverage-related artifacts, could generate the most comprehensive spectrum of mtDNA variants with satisfactory robustness.

To our knowledge, fNUMT is the first method that can simultaneously detect non-ref NUMTs and the derived non-ref NUMT-FPs, which is essential for investigating the interaction between nDNA and mtDNA and improving the accuracy of mtDNA variants detection. Regarding the non-ref NUMTs, the advantages of fNUMT over the current methods NUMTs-detection and dinumt in false positive rates and breakpoint precision lie in the conduction of local assembly of soft-clipped reads, coupled with the full consideration of their mapping direction [38, 39] The latter is essential for determining the start and end coordinates of the inserted mtDNA segments, which could otherwise be mistaken as 16,569 minus the true size since mtDNA is a circular molecular. Moreover, the direct output of non-ref NUMT-FPs by fNUMT could be filtered out from the initial mtDNA variants, which is time and cost-efficient.

The accumulation of the WGS data of 663 ESCC tumor-normal samples provides the opportunity for investigating mtDNA in ESCC. Moreover, the RNA-seq and WGBS data help reveal the underlying consequences of mtDNA alterations. The analysis of mtCN demonstrated the association between TN-ratio and overall survival in ESCC. Given the different metabolic basis among patients, this is more reasonable than previous studies that compared the mtCN of tumors among individuals [15]. The identification of the increases of TN-ratio with the progression of T stages, due to the mtCN increase in tumors, was consistent with the previous finding that mtCN increased as the disease progressed from noncancerous esophageal mucosa to ESCC and metastatic lymph nodes [25]. Further investigation of the differentially expressed genes and the enriched pathways suggested that tumor cells were probably more capable of striving for energy, escaping cell cycle surveillance, and DNA damage repair than the normal cells in the TN-ratio > 1 patients. These may help tumor cells to survive and adapt to the changing micro-environment during tumor initiation and progression, which results in poorer survival of the TN-ratio > 1 patients. Importantly, this finding is easily transformable in clinical practice to predict patient survival and help therapeutic decision-making since real-time PCR could efficiently measure the mtCN.

The analysis of non-ref NUMTs demonstrated a negative selection pressure of the large-size non-ref NUMTs in ESCC. Additionally, we observed the downregulation of *CBWD1* expression by a non-ref NUMT located on its intron region, which may subsequently affect ATP binding activity and provide conditions for tumor cells to adapt to a hypoxic environment. This non-ref NUMT is also present in the general population (46.8% of the East-Asian population, Additional File 2: Table S2). The reflection on the association of a common non-ref NUMT with ESCC would be interesting. However, further studies are needed to verify the detailed functional effect of this non-ref NUMT on ESCC.

The identification of low-heteroplasmic mtDNA variants provides enough power to predict the mutational signature whereby we observed a positive relationship between the number of truncating mutations with the contribution of signatures linked to aristolochic acid exposure, defective DNA base excision repair, and azathioprine and temozolomide treatment, suggesting the essential roles of mtDNA variants in tumorigenesis and treatment response. The variants with VAF > 0.01 showed the C > T and T > C transitions dominant mutation spectra consistent with a previous pan-cancer study [14]. In addition, we observed that the large percent T > G variants with VAF < 0.01, which recently occurred and not yet accumulated in cells, were associated with ESCC prognosis. This may portray a form of mtDNA instability, presented as a large number of low-frequent T > G variants, which may be relevant to the progress and prognosis of ESCC. Moreover, the mutational signature of mtDNA variants differs from that of nDNA highly linked to aging [23], highlighting the importance of studying mtDNA in extending the current understanding of ESCC etiology.

## Conclusions

This study proposes two newly developed methods to overcome the current challenge in characterizing the molecular features of mtDNA based on the short-read WGS data, with the potential to promote the research of mtDNA in human diseases. In addition, the multi-omics profiling of mtDNA in ESCC, as well as the newly identified ESCC-associated mtDNA features, extend the current understanding of ESCC etiology and may pave the foundation for the early screening and therapeutic decision-making of ESCC.

### Supplementary Information

**Additional file 1: Fig. S1.** The performance comparison of four tools in detecting Indels. **Fig. S2.** The IGV plot of ten non-ref NUMTs. **Fig. S3.** The IGV plot of the most confident non-ref NUMT detected by NUMTs-detection. **Fig. S4.** The circos plots of non-ref NUMTs of different sizes and origins. **Fig. S5.** The IGV plot and the alignment of the inserted sequences extracted from the long-read sequencing data. **Fig. S6.** The filtering of variants inside the mtDNA NUMT segments. **Fig. S7.** The correlation between the number of variants per sample and mitochondrial depth under different VAFs before and after mtDNA copy number-based filter. **Fig. S8.** The 96 mutational contexts of SNVs of all samples. **Fig. S9.** The mutational spectrum of SNVs and the association with ESCC prognosis.

**Additional file 2: Table S1.** The non-ref NUMTs in two ESCC patients based on short-read and long-read sequencing data. **Table S2.** The 110 distinct non-ref NUMTs and the occurrence in the previous study. **Table S3.** The 126 ESCC tumor specific mtDNA variants.

## Data Availability

The datasets supporting the conclusions of this article are available in the Genome Sequence Archive (GSA) in the Beijing Institute of Genomics (BIG) Center, Chinese Academy of Sciences (https://ngdc.cncb.ac.cn/gsa-human/browse): WGS data of 508 paired samples (HRA000021) [23], WGS, RNA-seq, and WGBS data of 155 paired samples (HRA003107, HRA003533) [24], and the short-read and Nanopore WGS data of two paired samples (HRA002508) [47]. dMTLV is available from https://github.com/sunnyzxh/dMTLV [59]. fNUMT can be downloaded from https://github.com/sunnyzxh/fNUMT [60]. These tools were accessible under the GNU General Public License.

## Abbreviations

ATP: Providing adenosine triphosphate
COSMIC: Catalogue of Somatic Mutations in Cancer
EC: Esophageal carcinoma
ESCC: Esophageal squamous cell carcinoma
GATK: Genome Analysis Toolkit
GIV: Global imbalance value
HGDP: Human Genome Diversity Project
IGV: Integrated Genomics Viewer
mtCN: MtDNA copy number
mtDNA: Mitochondrial DNA
nDNA: Nuclear DNA
NMF: Non-negative matrix factorization
NUMTs: Nuclear sequences of mitochondrial origin
non-ref NUMTs: Non-reference NUMTs
non-ref NUMT-FPs: Non-reference NUMT derived false positives
PCR: Polymerase chain reaction
PPV: Positive predictive rate
rCRS: Revised Cambridge Reference Sequence
SBS: Single base substitution
SNVs: Single nucleotide variations
SVs: Structural variants
TPR: True positive rate
VAF: Variant allele fraction
WGBS: Whole-genome bisulfite sequencing
WGS: Whole-genome sequencing
1KGP: 1000 Genomes Project
