# Supplementary material for: Leveraging new methods for comprehensive characterization of mitochondrial DNA in esophageal squamous cell carcinoma
Source: Genome Med. 2024 Apr 2;16:50. doi: 10.1186/s13073-024-01319-2 (PMC10985887; doi:10.1186/s13073-024-01319-2)
Supplement: Supplementary file 1 — Additional file 1: Fig. S1. The performance comparison of four tools in detecting Indels. Fig. S2. The IGV plot of ten non-ref NUMTs. Fig. S3. The IGV plot of the most confident non-ref NUMT detected by NUMTs-detection. Fig. S4. The circos plots of non-ref NUMTs of different sizes and origins. Fig. S5. The IGV plot and the alignment of the inserted sequences extracted from the long-read sequencing data. Fig. S6. The filtering of variants inside the mtDNA NUMT segments. Fig. S7. The correlation between the number of variants per sample and mitochondrial depth under different VAFs before and after mtDNA copy number-based filter. Fig. S8. The 96 mutational contexts of SNVs of all samples. Fig. S9. The mutational spectrum of SNVs and the association with ESCC prognosis. [file 13073_2024_1319_MOESM1_ESM.docx]

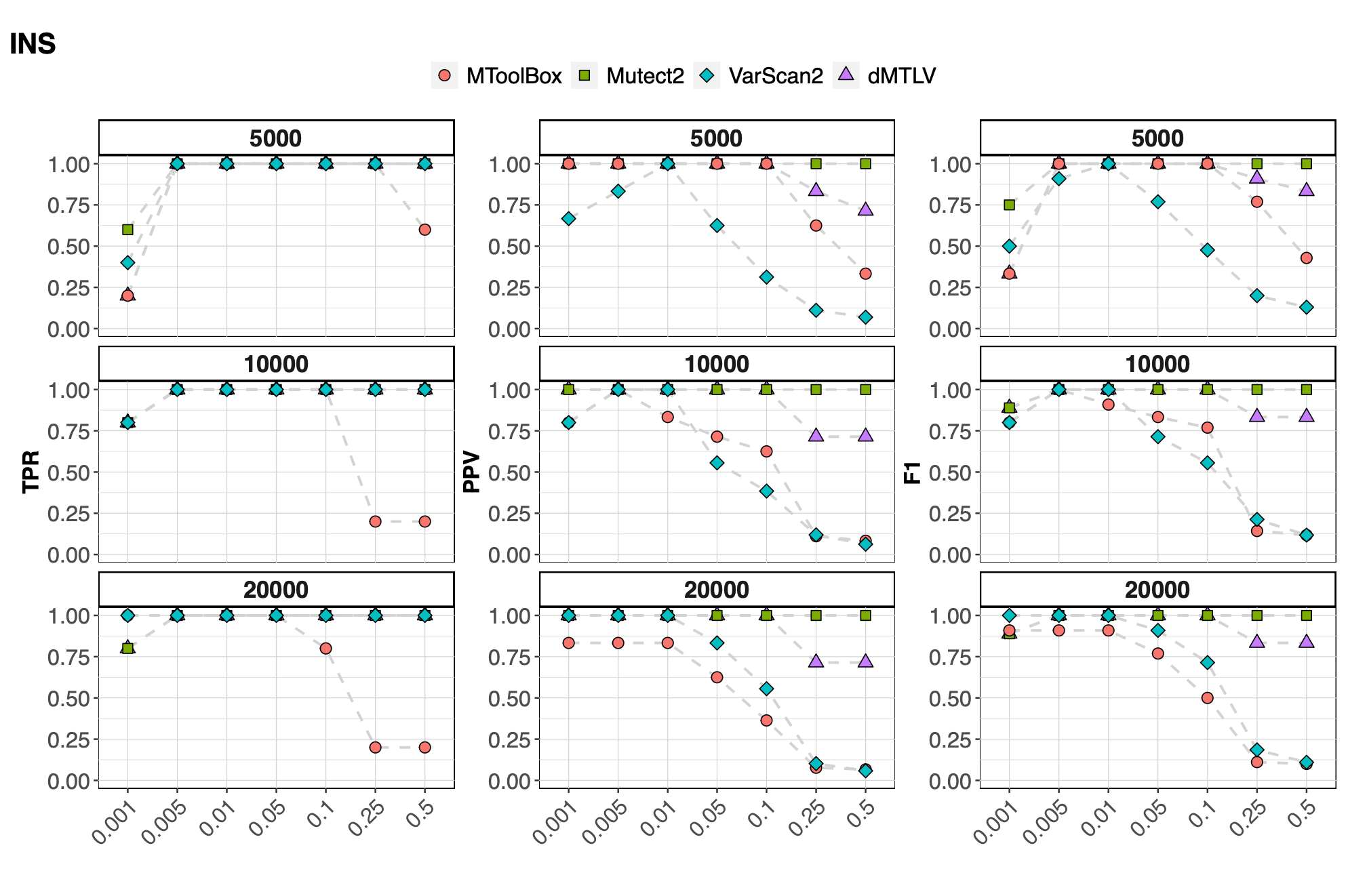

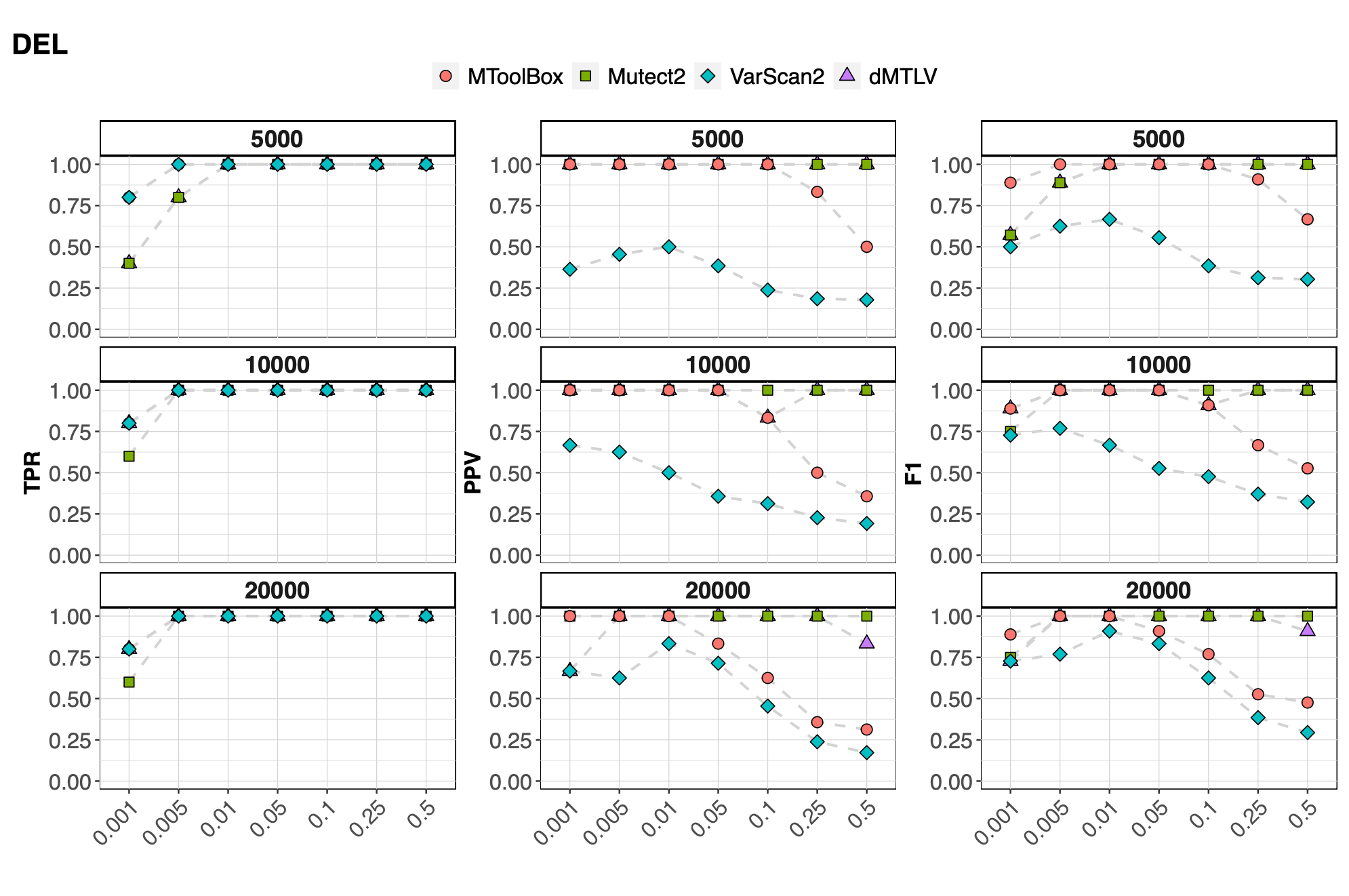


**Fig S1. The performance comparison of four tools in detecting Indels.** The true positive rate (TPR), positive predictive rate (PPV), and F1 of four tools: MToolBox, Mutect2, VarScan2, and dMTLV in detecting randomly simulated insertions (INS, top) and deletions (DEL, bottom) under seven distinct VAFs and three different coverages.


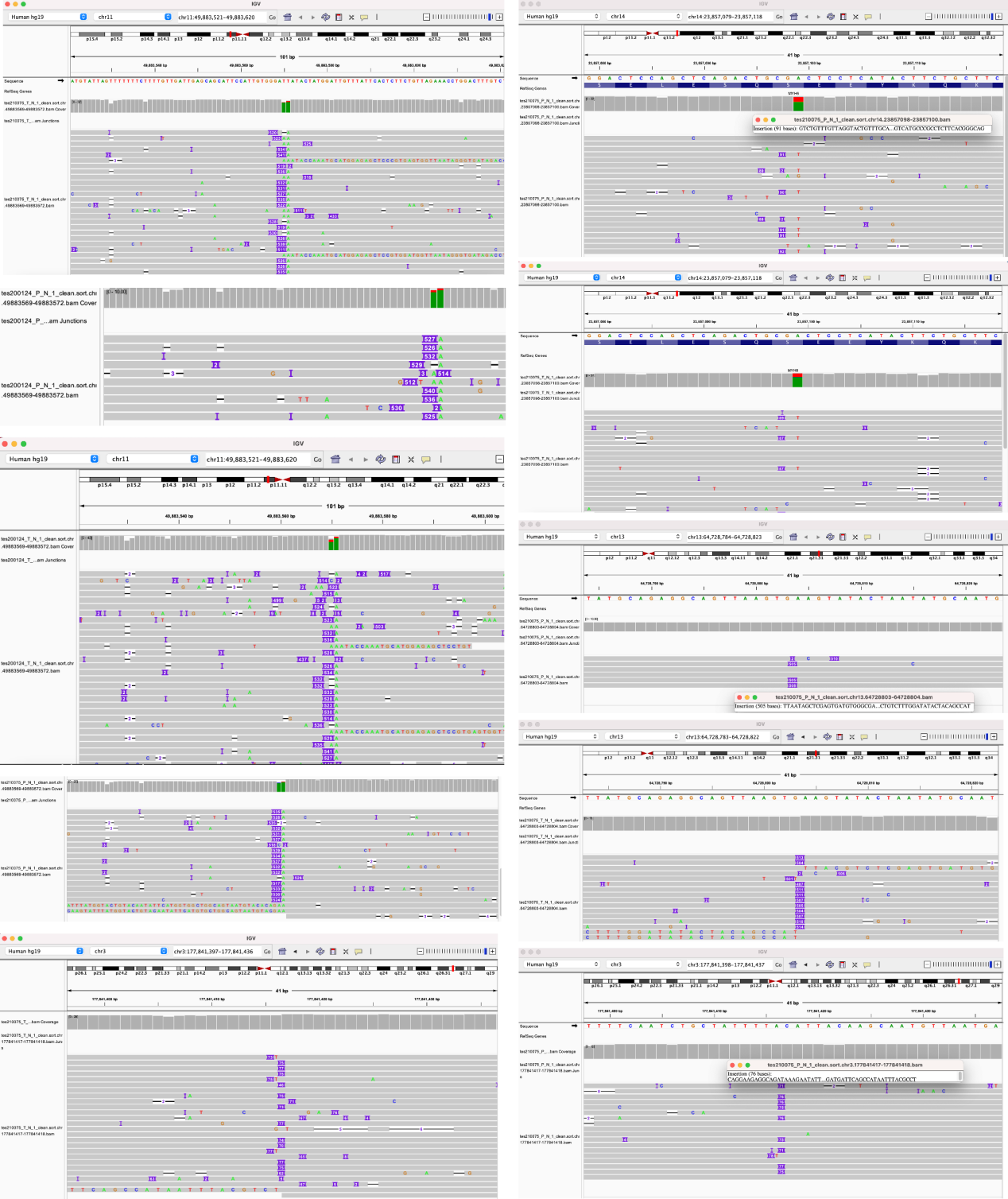


**Fig S2. The IGV plot of ten non-ref NUMTs.** The IGV plot of long-read sequencing data confirms the 10 non-reference NUMTs detected by fNUMT based on short-read sequencing data.

NUMT-detection


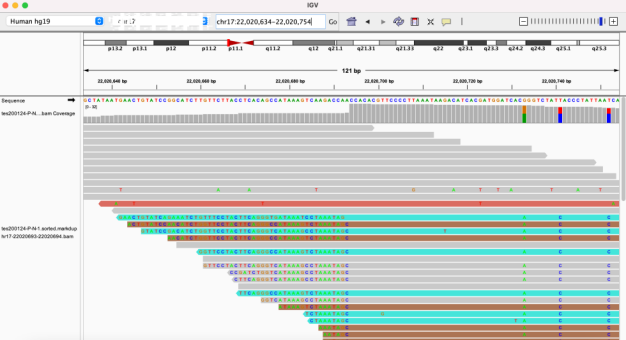

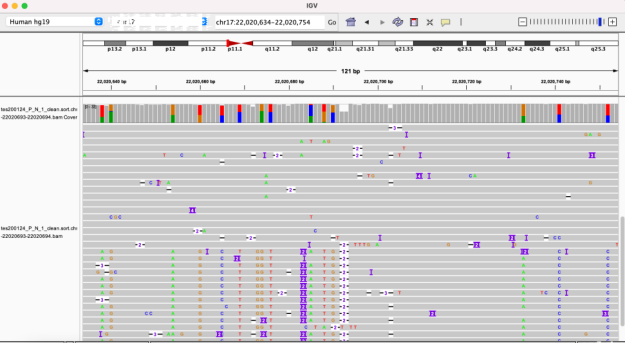


dinumt


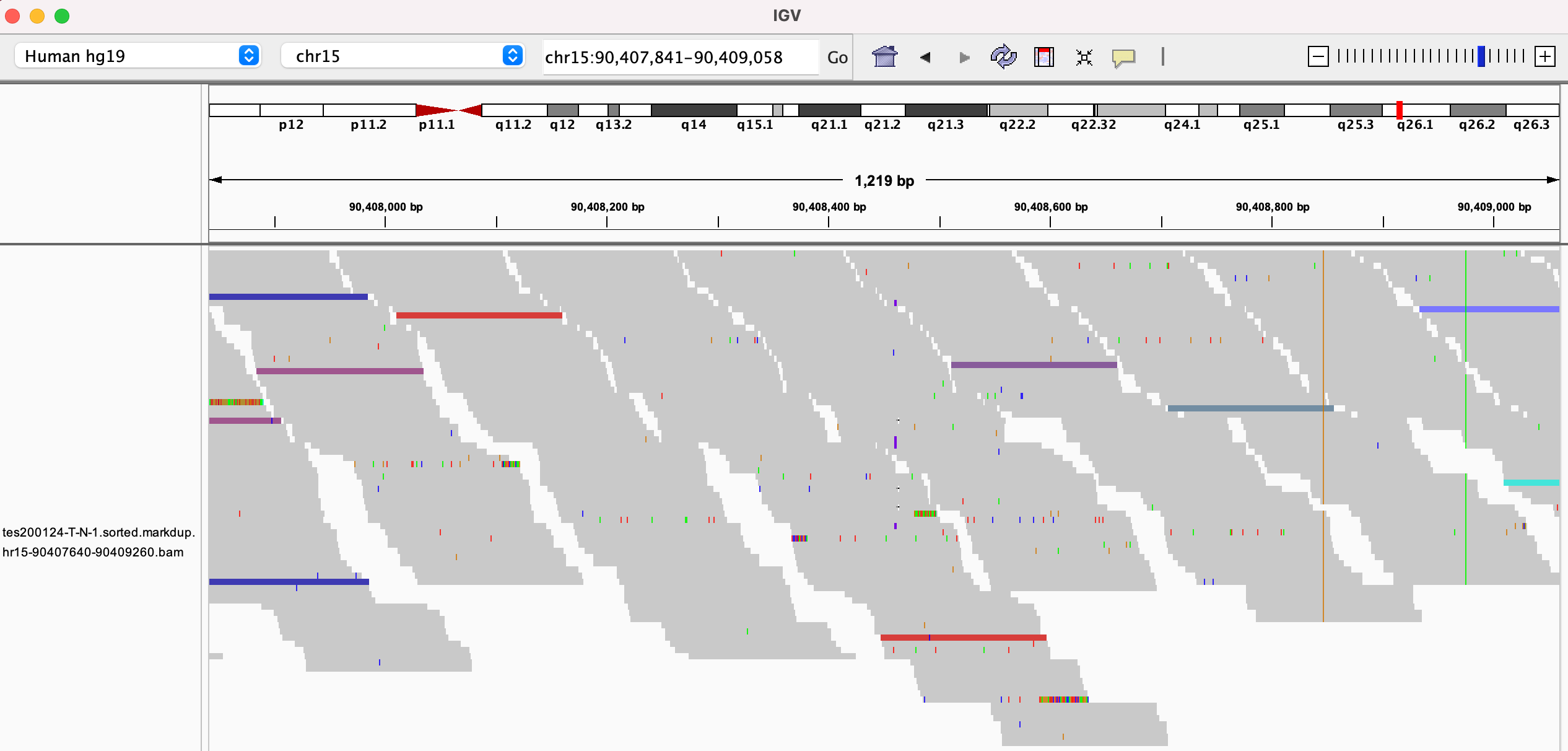

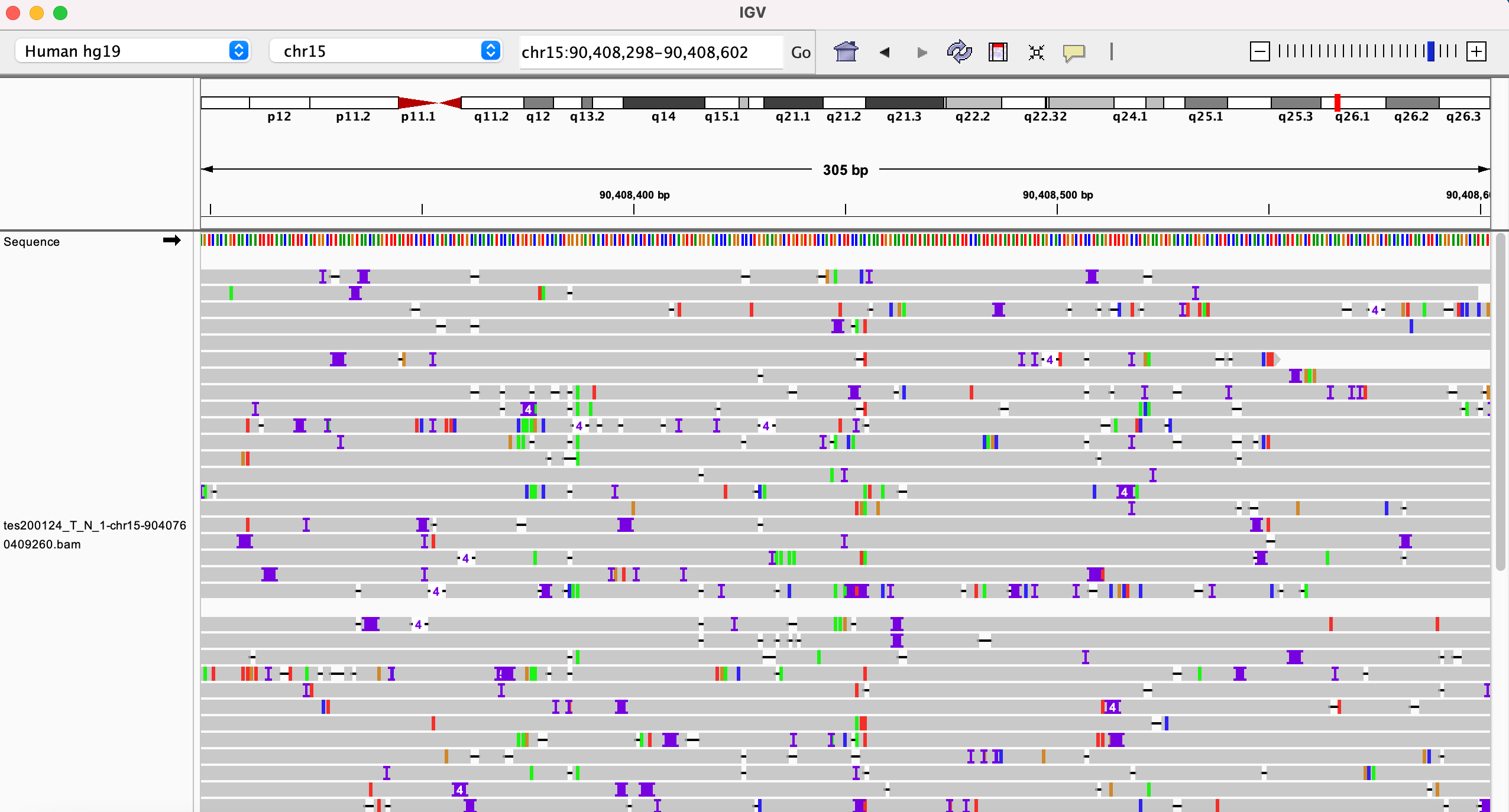


**Fig S3. IGV plot of the most confident non-ref NUMT detected by NUMTs-detection and dinumt.** left: short read; right: long read. These two non-ref NUMTs could not be verified by the long-read sequencing data.


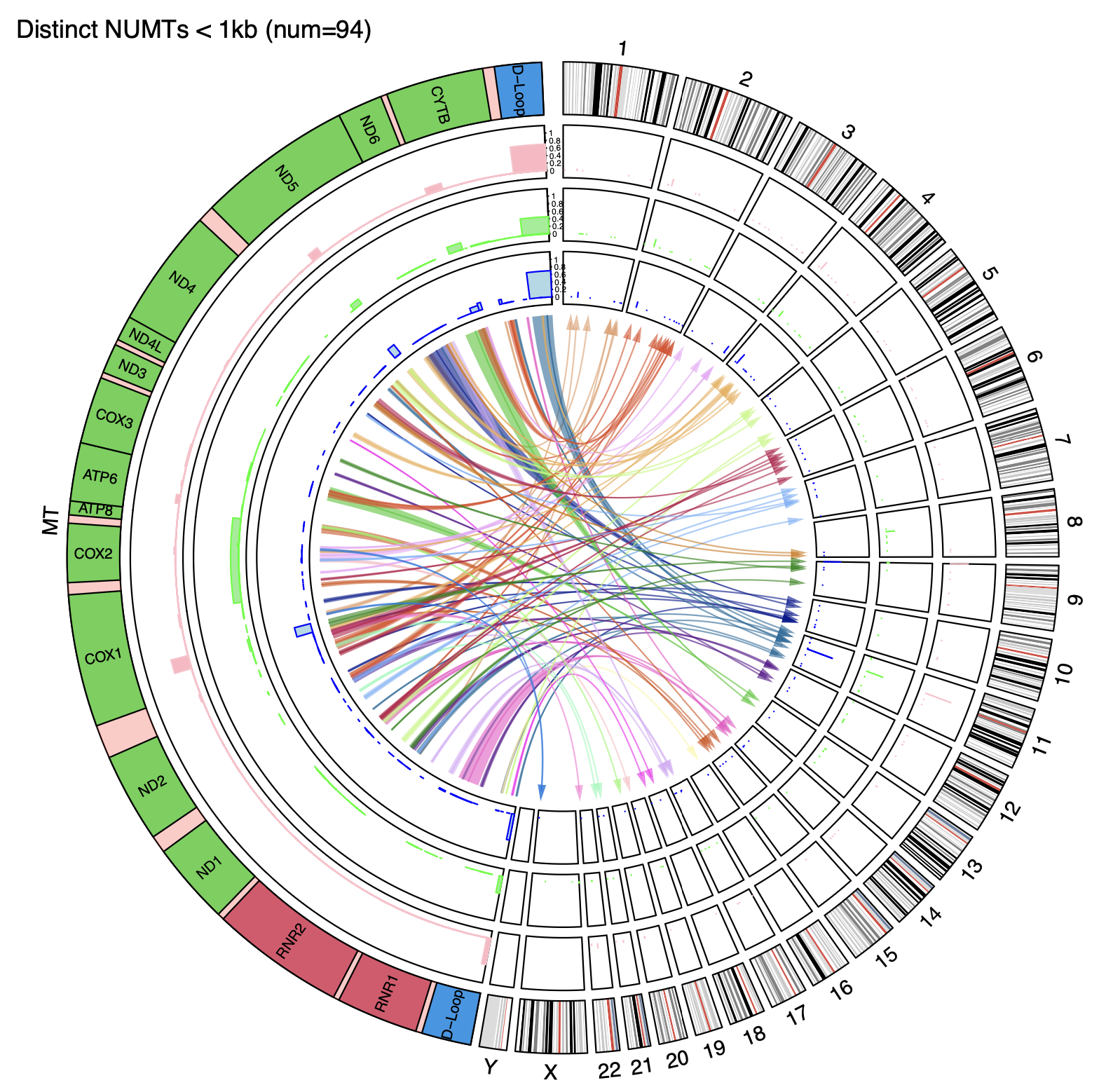

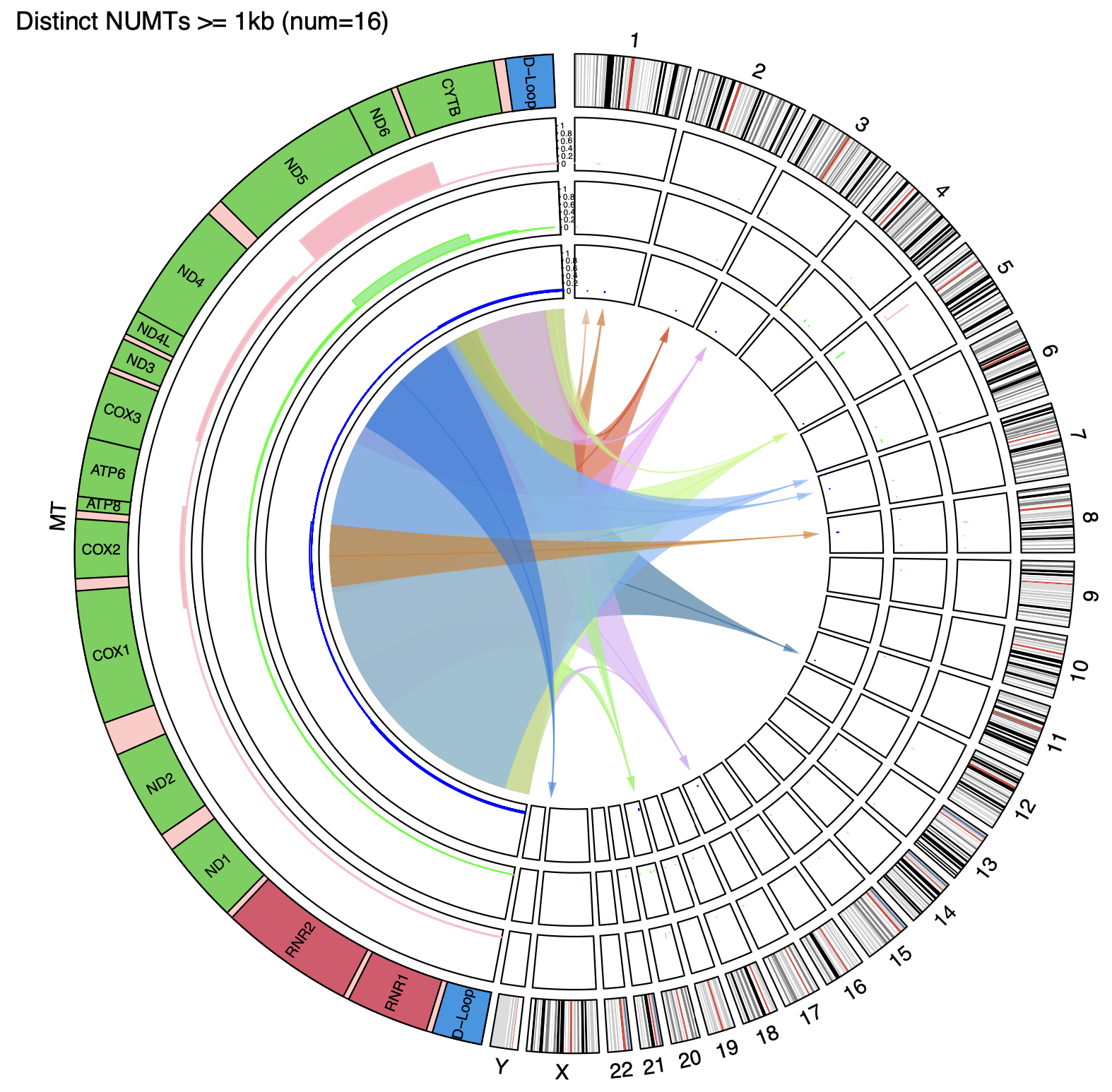


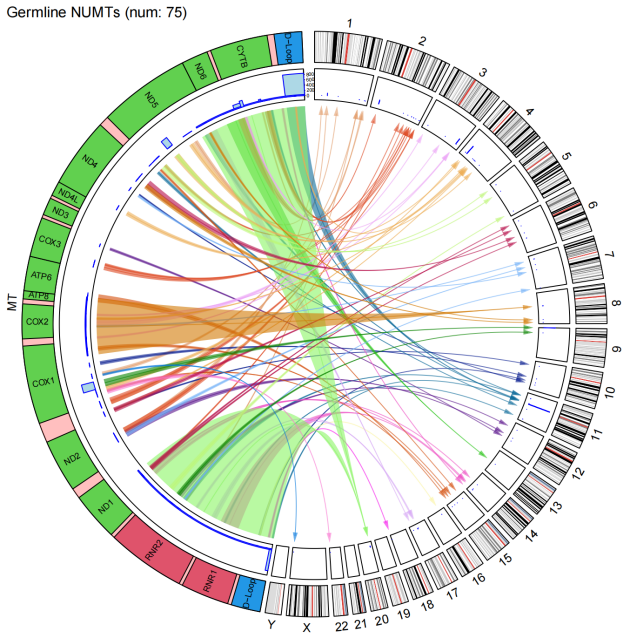

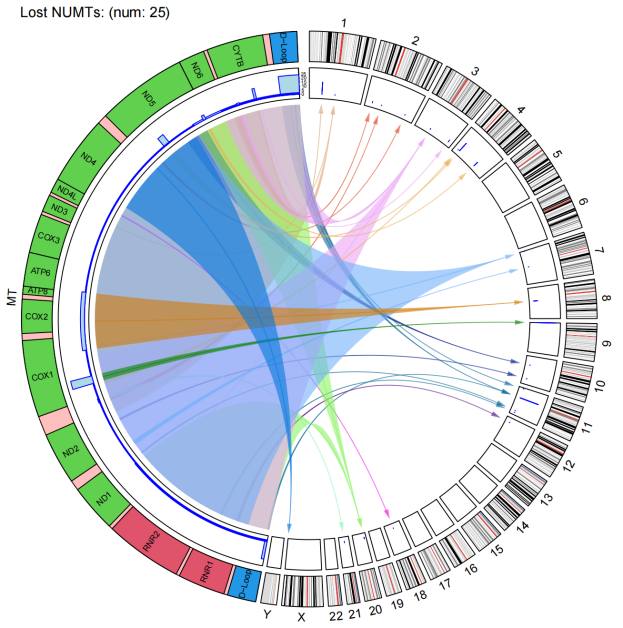


**Fig S4. The circos plots of non-ref NUMTs of different sizes and origins.** Top: From the outside: 1) mtDNA genes (left) and nDNA chromosome (right); 2) frequencies of non-ref NUMTs detected in Wei W. et al. [38]; 3) frequencies of non-ref NUMTs detected in Dayama G. et al. [39]; 4) frequencies of non-ref NUMTs detected in this study; 5) arrows representing the insertion of mtDNA segments to the breakpoints of nDNA for non-ref NUMTs detected in this study. Bottom: From the outside: 1) mtDNA genes (left) and nDNA chromosome (right); 2) frequencies of non-ref NUMTs detected in this study; 3) arrows representing the insertion of mtDNA segments to the breakpoints of nDNA.


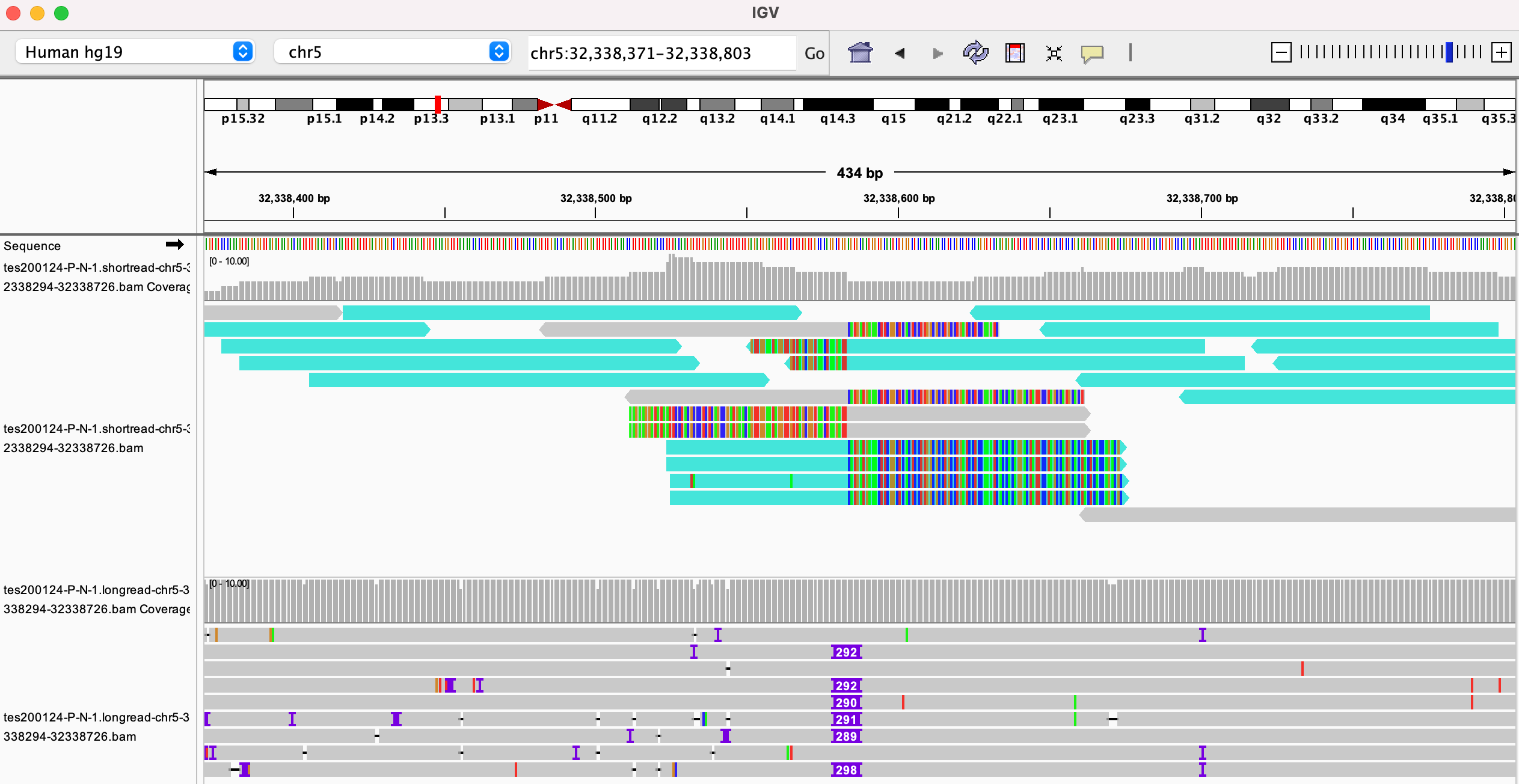


The inserted 292bp sequence extracted from long-read sequencing data:

CATGATGAAACTTCGGCTCACTCCTTGGCGCCTGCCTGATCCTCCAAATCACCACAGGACTATTCCTAGCCATGCACTACTCACCAGACGCCTCAACCGCCTTTTCATCAATCGCCCACATCACTCGAGACGTAAATTATGGCTGAGATACGGTTGTATAGGATTGCTTGAATGGCTGCTGTGTTGGCGTCTGCCGGGCGTATCATCAACTGGATGAGCAAGAAGGATATAATTCTACGCCCTCTCAGCCGATGAACAGTTGGAATAGGTTGTTAGCGGTAACTAAGATT

blast result:


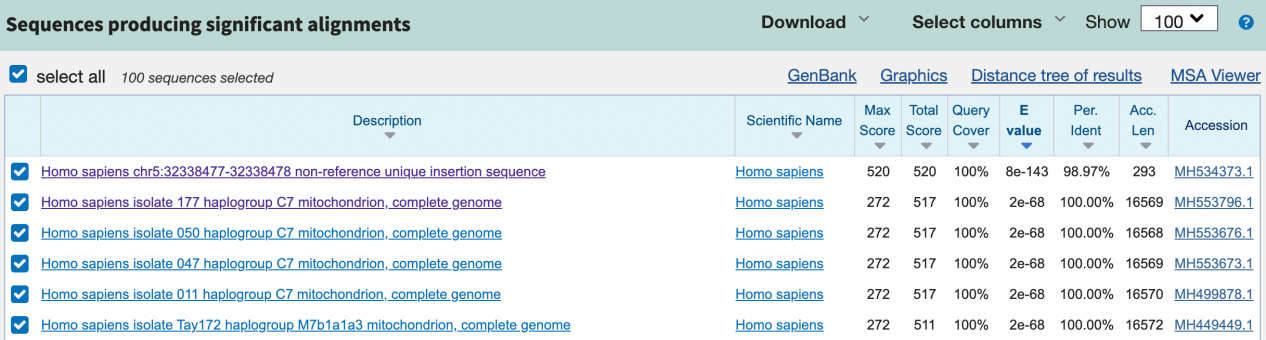


the best hit:


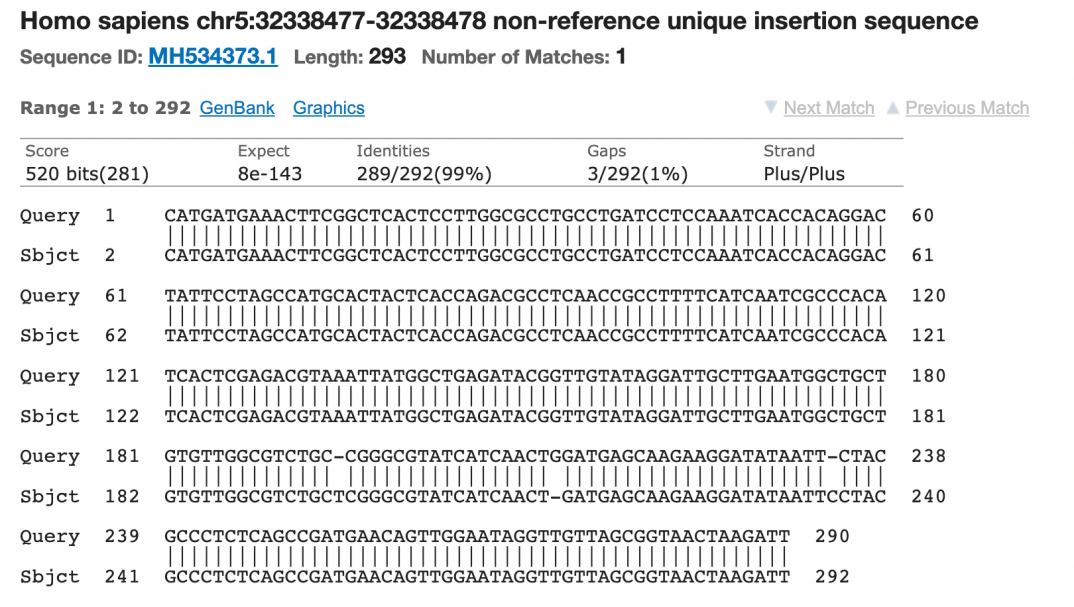


the second best hit:


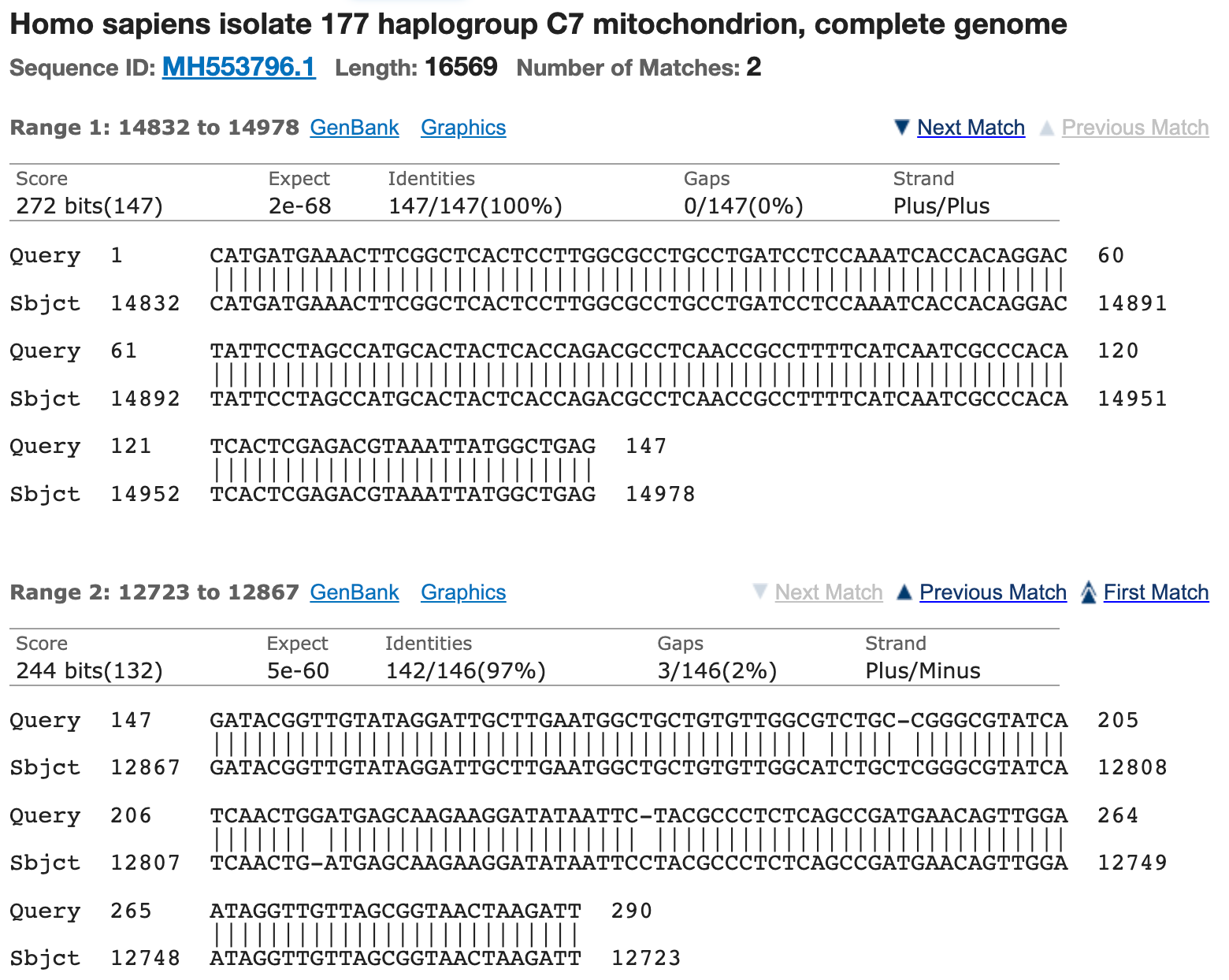


**Fig S5. The IGV plot and the alignment of the inserted sequences extracted from the long-read sequencing data.** The result demonstrated the false positive of the non-ref NUMT chrM:12714-14830-to-chr5:32338477 detected by NUMTs-detection.


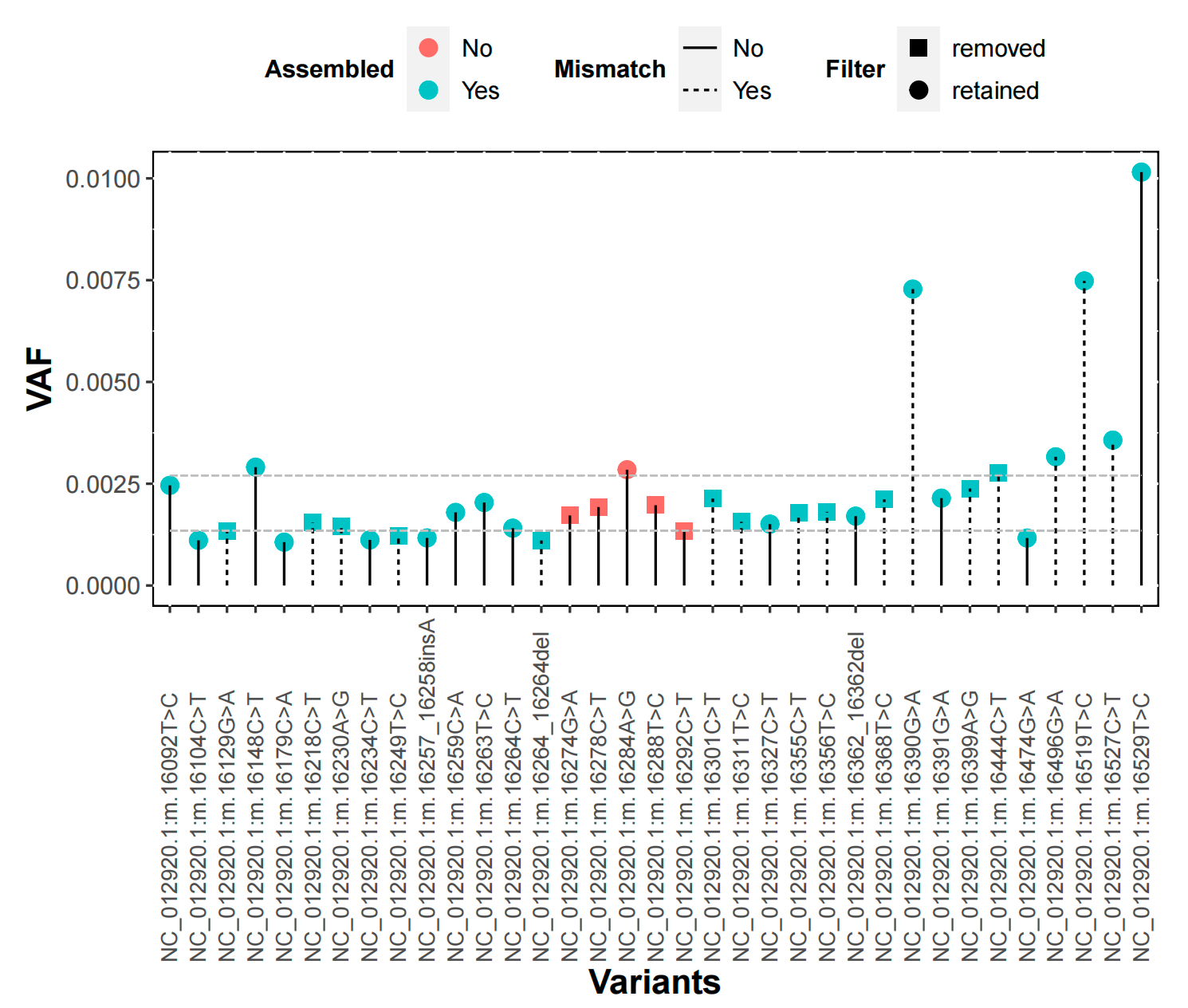


**Fig S6. The filtering of variants inside the mtDNA NUMT segments.** Take one sample as an example.


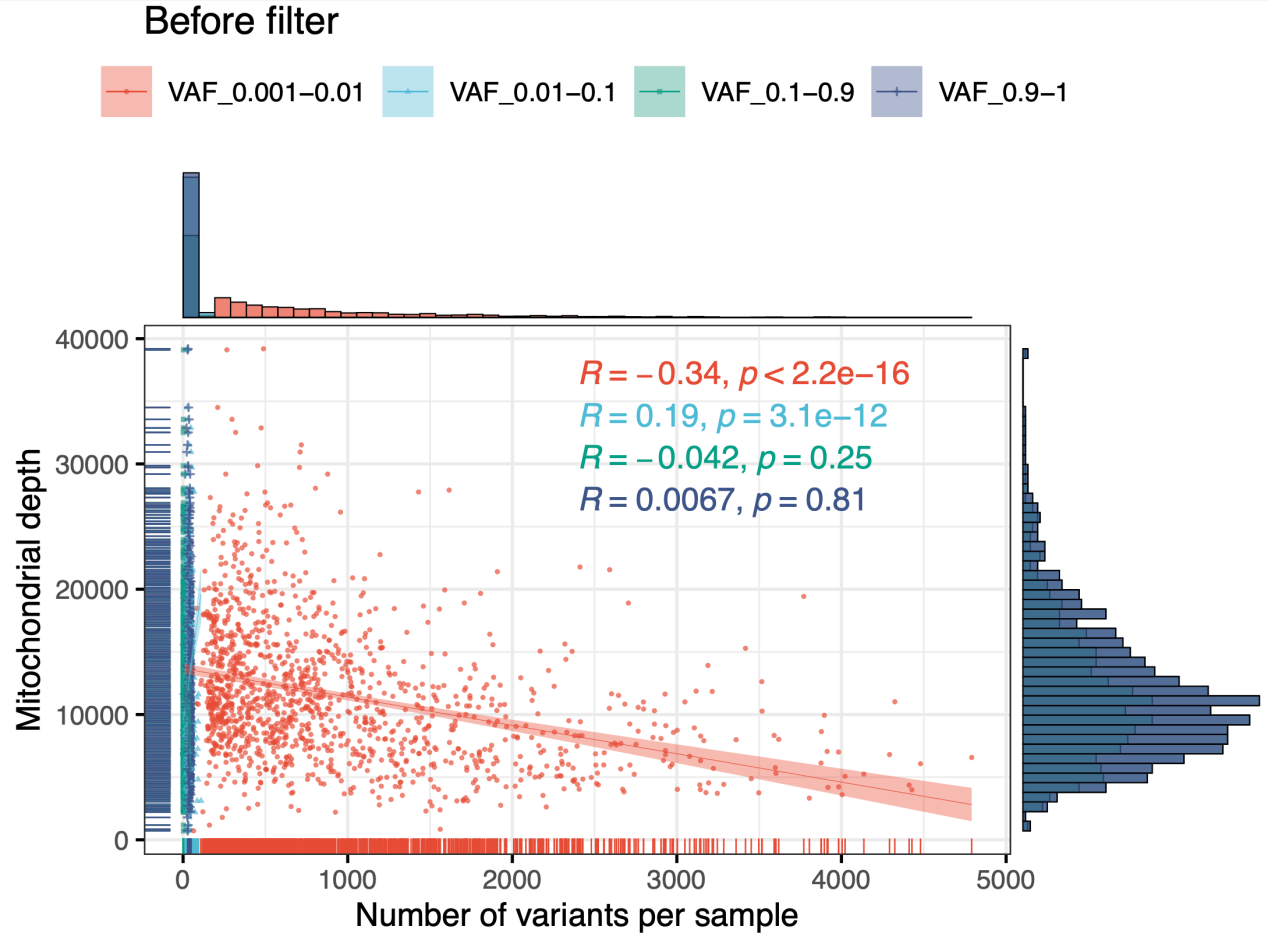


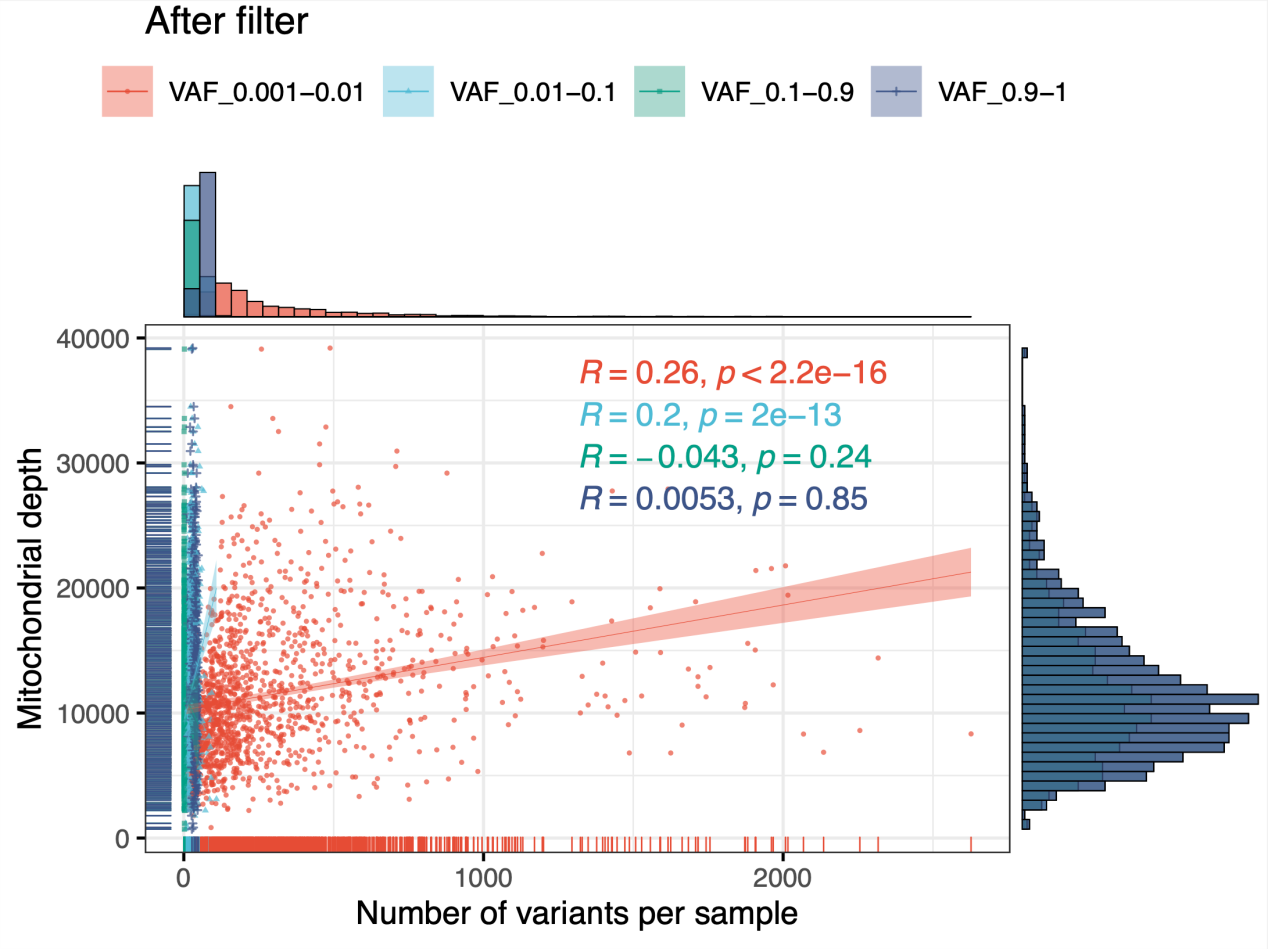


**Fig S7. The correlation between the number of variants per sample and mitochondrial depth under different VAFs before and after mtDNA copy number-based filter.**


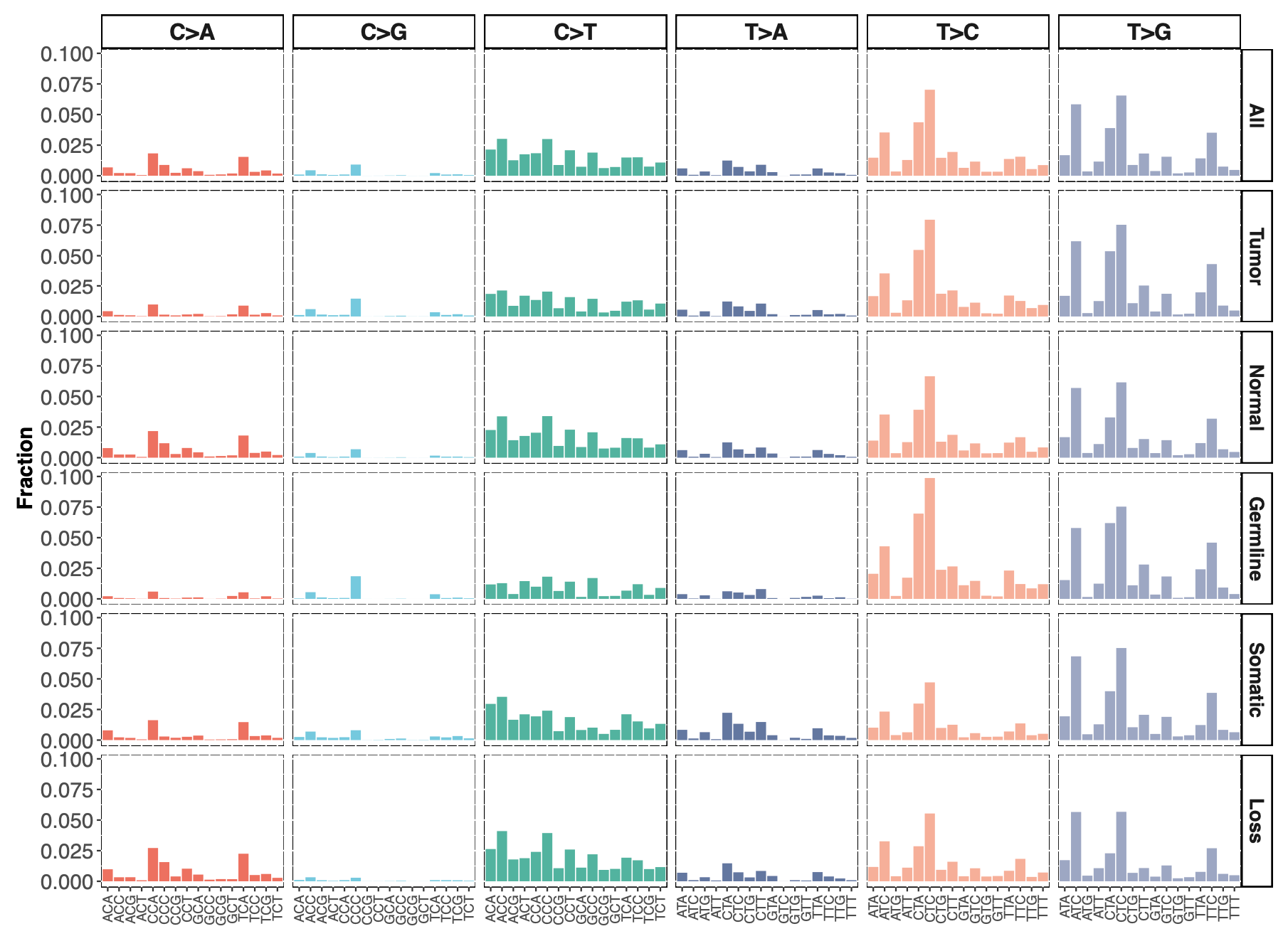


**Fig S8. The 96 mutational contexts of SNVs of all samples.** The result was grouped by sample types (tumor and normal), and by the source of variants (germline, somatic, and loss).


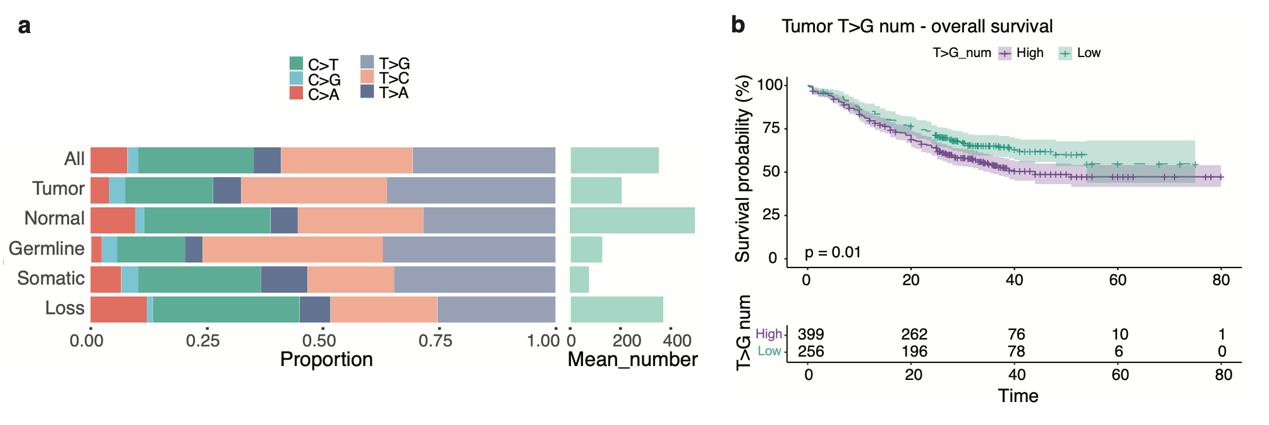


**Fig S9. The mutational spectrum of SNVs and the association with ESCC prognosis.** a: The proportion of six mutational types of SNVs of all, tumor and normal samples, and different sources of the germline, somatic, and loss mtDNA variants. The bar plot of the mean number of mtDNA variants per type is shown on the right. b: Plots of overall survival for patients with high and low numbers of T>G/A>C mutations with VAF<0.01 in tumor samples. The colored areas indicate the 95% confidence intervals, with the risk table under the survival plot. P value was measured by log-rank test.
